# Reasoning-Driven Design of Single-Atom Catalysts via a Multiagent Large Language Model Framework

**DOI:** 10.1021/acscentsci.6c01260

**Published:** 2026-09-04

**Authors:** Dong Hyeon Mok, Seoin Back, Victor Fung, Guoxiang Hu

**Affiliations:** † Department of Chemical and Biomolecular Engineering, Institute of Emergent Materials, 35014 Sogang University, Seoul 04107, Republic of Korea; ‡ KU-KIST Graduate School of Converging Science and Technology, 34973 Korea University, Seoul 02841, Republic of Korea; § Department of Integrative Energy Engineering, 34973 Korea University, Seoul 02841, Republic of Korea; ∥ Institute for Multiscale Matter and Systems (IMMS), Ewha Womans University, Seoul 03760, Republic of Korea; ⊥ School of Computational Science and Engineering, 1372 Georgia Institute of Technology, Atlanta, Georgia 30332, United States; # School of Materials Science and Engineering, 1372 Georgia Institute of Technology, Atlanta, Georgia 30332, United States; ∇ School of Chemistry and Biochemistry, 1372 Georgia Institute of Technology, Atlanta, Georgia 30332, United States

## Abstract

Large language models (LLMs) are becoming increasingly applied beyond natural language processing, demonstrating strong capabilities in complex scientific tasks that traditionally require human expertise. This progress has extended into materials discovery, where LLMs introduce a new paradigm by leveraging reasoning and in-context learning, capabilities absent from conventional machine learning approaches. Here, we present a Multi-Agent-based Electrocatalyst Search Through Reasoning and Optimization (MAESTRO) framework in which multiple LLMs with specialized roles collaboratively discover high-performance single-atom catalysts for the oxygen reduction reaction. Within an autonomous design loop, agents iteratively reason, propose modifications, reflect on results, and accumulate design history. Through in-context learning enabled by this iterative process, MAESTRO identified design principles not explicitly encoded in the LLMs’ background knowledge and successfully discovered catalysts that break conventional scaling relations between reaction intermediates. These results highlight the potential of multiagent LLM frameworks as a powerful strategy to generate chemical insight and discover promising catalysts.

## Graphical abstract

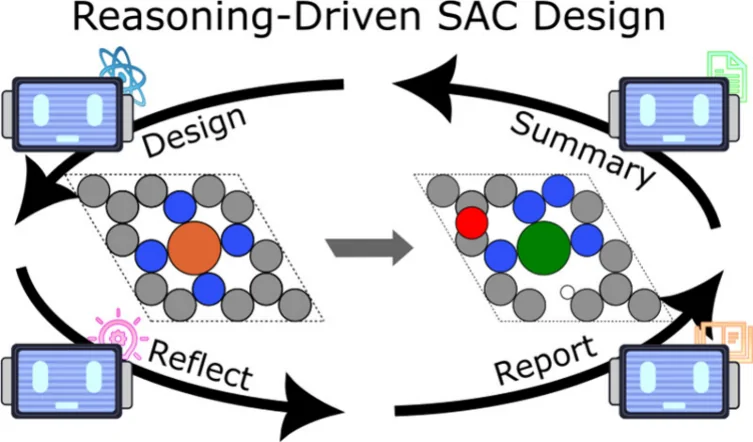

## 1 Introduction

Identifying new materials that deliver high catalytic activity, selectivity, and long-term stability has remained a significant and ongoing scientific challenge, with major implications for improving energy efficiency, lowering industrial costs, and enabling more sustainable chemical processes. Over time, materials discovery strategies have evolved beyond purely experimental approaches and first-principles simulations, such as density functional theory (DFT) calculations,^1–3^ with machine learning (ML) becoming one of the dominant paradigms in the field.^4–9^ Early ML-based approaches primarily relied on high-throughput screening,^10–13^ in which large data sets were generated in advance and property prediction models were used to filter promising candidates, thereby reducing the computational and experimental cost of subsequent validation. However, as the chemical search space expanded and performance requirements became increasingly demanding, such screening-based strategies encountered limitations related to data availability, computational cost, and scalability.^14,15^ To address these challenges, inverse design strategies were introduced, in which materials are designed starting from target properties rather than discovered through forward searches.^16–19^ Approaches based on global optimization^20,21^ and generative models^22–24^ have demonstrated notable success in the efficient discovery of promising materials.

Despite these advances, most data-driven methods remain dependent on the coverage and quality of pre-existing training data sets,^25,26^ which must typically be generated through experiments or first-principles calculations before model development. Their predictive reliability can deteriorate under distribution shift, limiting robust extrapolation to chemical or structural regimes that are poorly represented in the training data. Recent physics-informed and interpretable machine learning approaches have partially addressed these limitations by incorporating physical constraints or extracting chemically meaningful descriptors and mechanistic insight.^27,28^ However, such capabilities have not yet been integrated into end-to-end inverse design workflows in a manner that simultaneously proposes candidates and formulates explicit, transferable design hypotheses. Consequently, substantial human interpretation is still often required to translate model predictions into generalizable physical design principles.

Recent advances in large language models (LLMs) offer a fundamentally different avenue for inverse design. Originally developed for natural language processing, LLMs trained on massive text corpora have demonstrated a capacity for human-like reasoning.^29–31^ In chemistry and materials science, LLMs have begun to move beyond simple data mining toward roles involving experimental planning,^32^ synthesis guidance,^33^ simulation tool orchestration^34^ and chemically informed decision-making tasks that were previously exclusive to human experts.^35–38^ Crucially, LLMs possess the capability for in-context learning, whereby they adapt their reasoning based on prior interactions and accumulated context without explicit parameter updates.^39^ This enables LLMs to acquire task-specific principles and uncover new insights not explicitly encoded in their background knowledge, particularly when embedded within iterative decision-making loops.^40^ Furthermore, recent research has shifted from assigning multiple roles to a single LLM toward multiagent frameworks, whereby several LLM-based agents, each specialized for a specific task, interact and collaborate.^41,42^ Such systems provide a natural foundation for complex scientific workflows that require hypothesis formulation, reflection and memory.

In this work, we investigate whether an LLM-based multiagent framework can be successfully applied to heterogeneous electrocatalysis, a relatively narrow yet intrinsically complex domain. While LLMs have recently been employed in catalyst-related studies for tasks such as data mining,^43^ synthesis planning,^44^ property prediction^45^ and catalyst generation,^46^ approaches that explicitly leverage LLM reasoning to design high-performance catalysts remain scarce compared to other systems. To address this gap, we develop a Multi-Agent-based Electrocatalyst Search Through Reasoning and Optimization (MAESTRO) framework in which multiple LLM agents collectively design active and stable single atom catalysts (SACs) for the oxygen reduction reaction (ORR) (Figure 1). Within this framework, agents iteratively propose structural modifications, reflect on outcomes and summarize design history, while rapid property evaluation is enabled by a machine learning force field (MLFF) serving as a surrogate for DFT.^47^

**Figure 1:**
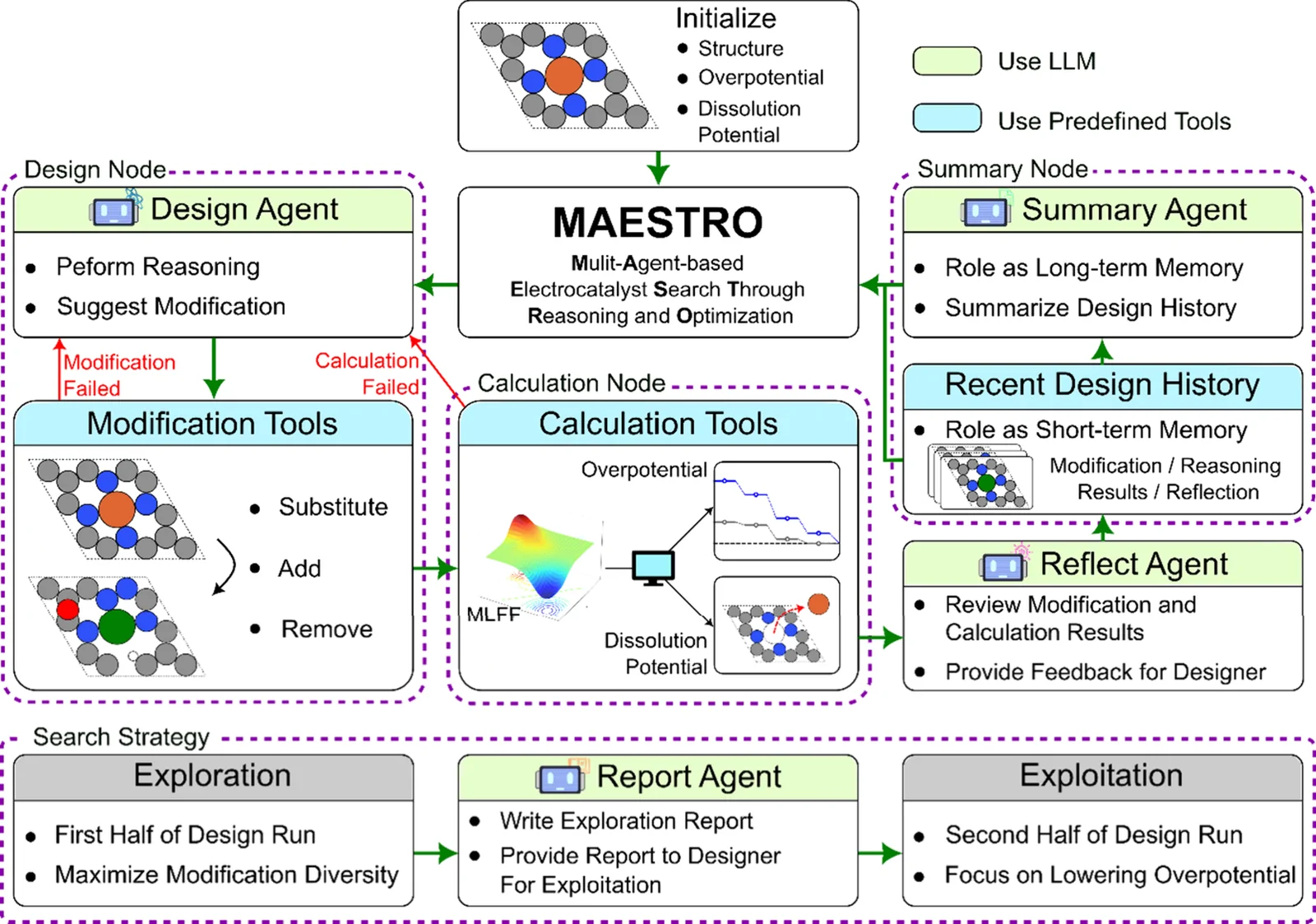
Overview of the MAESTRO framework and the exploration–-exploitation search strategy. Starting from an initial catalyst structure and its associated properties, LLM-based agents and predefined tools iteratively perform reasoning, modification, evaluation, reflection and summarization according to their designated roles for a fixed number of cycles. The design run is divided into two phases, an exploration phase and an exploitation phase. Upon completion of the exploration phase, a summary report is generated by the agent.

Applying the MAESTRO framework, we demonstrate that the agents can autonomously formulate hypotheses regarding catalyst modification strategies and progressively improve both activity and stability through iterative reasoning. Notably, the framework identifies SACs that surpass the theoretical activity limit imposed by conventional scaling relations between reaction intermediates.^48^ Detailed analysis reveals that this enhancement originates from the selective stabilization of a specific intermediate via hydrogen bonding, a mechanism previously reported in several studies. Importantly, this discovery does not emerge in the absence of in-context learning, providing strong evidence that the proposed framework enables agents to acquire new physics and design principles through accumulated history and iterative reasoning. These results highlight the MAESTRO framework as a promising optimization strategy for electrocatalyst discovery, capable of navigating complex design spaces and uncovering nontrivial relationships between structure and property beyond conventional discovery schemes.

## 2 Results

### 2.1 MAESTRO Framework

The MAESTRO framework operates as an iterative design loop composed of four nodes, design, calculation, reflection and summary, managed by four agents, Design, Reflect, Summary and Exploration report, along with predefined tools. The primary objective of this loop is to minimize the ORR overpotential (η), which serves as a measure of the catalytic activity of the SAC, while simultaneously maximizing the dissolution potential (*U*_*diss*_) to ensure electrochemical stability.

The design loop initiates by loading an initial SAC structure and evaluating its overpotential and dissolution potential using a MLFF surrogate model. The resulting geometric and catalytic data are then formatted and passed to the design agent within the design node. Leveraging structural images and formatted metadata, the design agent formulates a hypothesis to identify specific modifications, and the underlying physical reasons, that could effectively tune binding energies to reduce the overpotential. The design agent is authorized to modify five distinct geometric components of the SAC: the center metal atom, first coordination shell, second shell, axial ligand and functional groups. The available modification types and options are summarized in Table 1.

**Table 1:** Geometric Components of the SAC Accessible to the Design Agent, Including Modification Types and Available Chemical Options^a^

Geometric Components	Modification Types	Atom/Molecule Options
Center metal atom	Substitute	Pt, Pd, Ir, Ru, Fe, Co, Mn, Cu, Ni, Cr, V, Ti, Mo, Na, Ta, Ag, Au, Zn, Sn, Bi
First coordination shell	Substitute, Add, Remove	H, C, O, N, S
Second coordination shell	Substitute	C, P, B, S, N
Axial ligand	Substitute, Add, Remove	*O, *OH
Functional group	Substitute, Add, Remove	*COC, *COH, *CO

a Specifically, axial ligands are introduced perpendicular to the surface, positioned beneath the binding site, while functional groups are added to the second coordination shell of the carbon support.

The proposed modification is forwarded to the modification tool, which verifies whether the suggested change is applicable to the given SAC. If the modification is deemed unsuitable, the workflow returns to the design agent for a new proposal. This includes cases in which the agent proposes invalid modifications, such as hallucinated modification type not included in the list or attempts to substitute nonexistent elements. If accepted, the modification is applied, and the resulting structure is passed to the calculation node. Here, the overpotential and dissolution potential of the modified SAC are calculated using the MLFF. If the geometry optimization fails to converge within 100 optimization steps under a force threshold of 0.05 eV/Å, the failed structure and associated metadata are returned to the design agent. Upon successful convergence, the calculated properties are forwarded to the reflection node.

In the reflection node, the reflection agent evaluates the effectiveness of the proposed modification by comparing the catalytic activity and stability before and after the change. Based on this comparison, it provides feedback categorizing the modification as successful or unsuccessful. This feedback, along with the proposed modification, the underlying reasoning, and the calculated results, is passed to the summary node and formatted as design history. While the most recent history is stored in full, previous entries are condensed by the summary agent to maintain context efficiency (Figure S2). Finally, the accumulated design history, the modified SAC structure and its performance metrics are fed back to the design agent to initiate the next iteration.

To ensure sufficiently broad exploration, the agents are guided by an exploration-exploitation strategy. During the first half of the process, the exploration phase, the primary objective is to expand the design space rather than minimize the overpotential. Accordingly, the design agent is instructed to propose structurally diverse and distinct types of modifications without prioritizing improvements in overpotential or dissolution potential. The reflect agent provides feedback which prioritizes the discovery of unique structural configurations.

At the midpoint of the design run, the workflow transitions to the exploitation phase, where optimization proceeds under the original objective of minimizing overpotential while maintaining stability. During this transition, an exploration report agent generates a one- to two-page report summarizing the modifications explored and their corresponding effects (Figure S7). This report distills actionable insights to guide the subsequent optimization. The exploration report serves as a persistent reference for the design agent throughout the remainder of the loop.

In this study, the primary results were obtained using GPT-4.1 mini as the LLM,^31^ Universal Models for Atoms (UMA) as the MLFF surrogate model,^49^ and FeN_4_ as the initial catalyst structure. However, the framework is modular and additional experiments using alternative models and starting materials were also conducted. Detailed agent prompts, catalyst specifications and implementation details are provided in Supplementary Note A.

### 2.2 Validation of MLFF and LLM Components

Before deploying the MAESTRO framework, we conducted a prevalidation study to ensure the reliability of both the tools and the LLM for the SAC system. Since the UMA employed as the MLFF in this study was not trained on SAC data, and no publicly available SAC data set existed for meaningful fine-tuning, we constructed a custom SAC data set to validate the extrapolation capabilities of UMA. The performance of the pretrained UMA was evaluated using this out-of-distribution (OOD) data set, which contains DFT calculated energies, forces and binding energies for various structures and their adsorbed intermediates. As shown in Figures 2a and 2b, UMA with ‘OC20’ domain showed Mean Absolute Errors (MAEs) for DFT energies per atom and atomic forces of 8.83 meV/atom and 47.73 meV/Å, respectively. These values are comparable to the MAEs reported by Wood et al. for OOD catalysts^49^ (Figure S3). For binding energy, the MAE of 0.346 eV is non-negligible and could introduce meaningful uncertainty if the MLFF values were used for screening based solely on absolute energetics. However, the primary objective of this study is not to establish UMA as a quantitatively exact replacement for DFT, but to examine whether LLM-guided structural modifications can steer binding energies in the intended direction, whether in-context learning improves the quality of such reasoning, and whether the iterative design loop can approach the desired optimum. In this context, even if the potential energy surface described by the MLFF proxy differs from that of DFT, the proxy can still provide a meaningful approximation of DFT-based design behavior as long as it preserves the relative trends in energy changes induced by candidate modifications. The interpretation is further supported by the largely systematic direction of the errors, which suggests that the model retains useful rank-level information despite its absolute offset. To explicitly evaluate this point, we conducted an additional binding energy validation using SAC structures generated from diverse modifications and found that the MLFF-predicted binding energies exhibited a high Spearman rank correlation with the corresponding DFT value, with ρ = 0.9698. These results support the use of UMA as a trend-preserving surrogate model for DFT within the design loop. Detailed information regarding the prevalidation data sets and MLFF performance is provided in Supplementary Note B.

**Figure 2:**
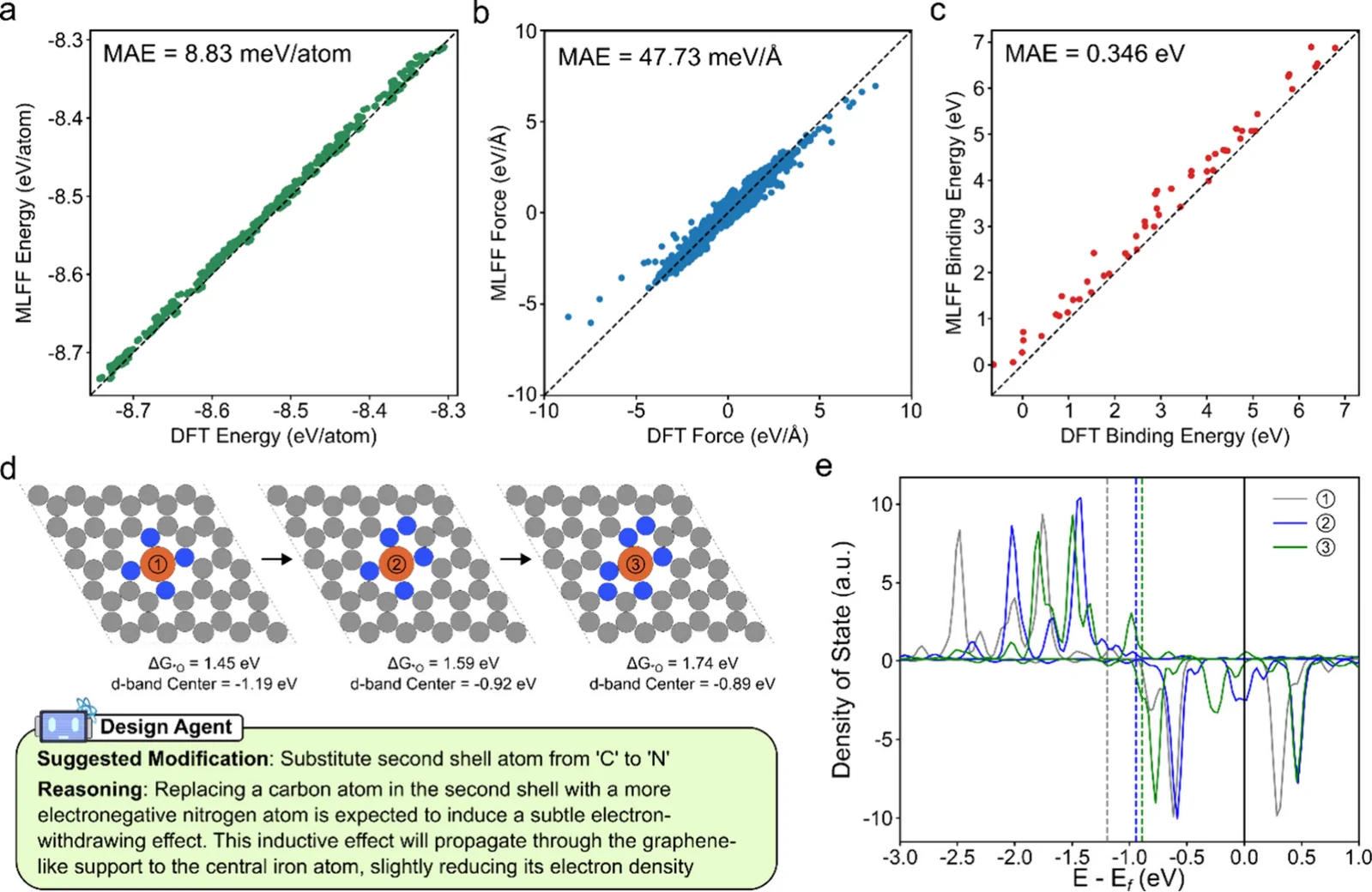
Prevalidation of MLFF and LLM components. (a–c) Parity plots comparing DFT-calculation and MLFF-prediction results for (a) energies per atom, (b) atomic forces and (c) binding energies. (d) An example of the reasoning and modification proposed by the design agent, together with the resulting structure and the changes in Gibbs free energy and d-band center induced by the suggested modification. (e) Corresponding changes in the electronic density of states (DOS) following the modification. Dashed lines indicate the d-band centers of the metal atoms, which shift toward positive values (closer to the Fermi level) upon the substitution of a second-shell carbon atom with nitrogen.

Regarding the LLM, we validated whether the modification proposed by the model could effectively steer binding energies in the intended direction and whether the underlying reasoning was physically sound. The target was to achieve a binding energy of 2.46 eV for the *O intermediate. As shown in Figure 2d, the LLM successfully suggested a modification that shifted the *O binding energy from the initial structure toward the desired target. Specifically, the LLM hypothesized that N substitution would decrease the electron density of the center metal atom. This hypothesis was confirmed by density of states (DOS) calculations, which showed a clear shift of electronic density, quantified by the reduction of d-band center position, of the Fe center after substitution.^50,51^ While the correspondence between the intended and realized binding energy changes and their associated reasoning was not perfect in every case, it was correct in the majority of instances. Representative examples of unsuccessful suggestions are provided in Figure S6.

These prevalidation results demonstrate that UMA can function as a practical surrogate for DFT in evaluating the stability and catalytic activity of SACs for ORR without additional fine-tuning. Moreover, the LLM demonstrates a meaningful understanding of structure–property relationships of the SACs, enabling it to propose chemically interpretable modifications that systematically guide the energies toward target values.

### 2.3 Performance Evaluation of Overall Framework

The objective of the design run is to minimize the ORR overpotential while simultaneously preserving or increasing the dissolution potential. To systematically evaluate the performance of our framework, we employed four metrics.

1) Average overpotential: The mean ORR overpotential obtained across all design runs, excluding the exploration phase. For strategies without an explicit exploration phase, this was computed over the latter half of each design run.
2) Minimum overpotential: The average of the lowest overpotential achieved during each individual design run.
3) Hypervolume: The average volume of the Pareto front, representing the joint optimization space spanned by limiting potential and dissolution potential. A larger volume indicates superior discovery performance in terms of both activity and stability.
4) Active-point volume: The average Pareto volume of the catalyst with the lowest overpotential, representing the combined activity and stability of the most promising candidate discovered.

To benchmark the performance of the proposed “*history + exploration*” strategy, which introduces an exploration phase to prioritize a global search before exploitation, we defined three baseline strategies: 1) The *history* strategy performs only exploitation without a preceding exploration phase, thereby isolating the effect of broadening the design space before focused optimization. 2) The *historyless* strategy relies solely on the LLM’s background knowledge and does not incorporate information from previous modification steps, highlighting the impact of in-context learning from accumulated design history. 3) The *random* strategy applies purely random modifications without LLM guidance, serving as a lower-bound reference.

Figure 3a and 3b present a representative design run, illustrating the gradual changes of overpotential and the corresponding Pareto front of the discovered catalysts. During the exploration phase, the overpotential exhibits large fluctuations as the framework broadly samples the design space. The exploratory behavior was further confirmed by the higher number of unique modifications performed during this phase, compared not only to the subsequent exploitation phase, but also to design runs of other strategies without exploration (Figure S8). In contrast, during the exploitation phase, the overpotential converges toward a narrow range, indicating focused optimization. The progression of the minimum overpotential, denoted by the red line and markers, reaches its optimum during this phase. The Pareto plot highlights the intrinsic trade-off between activity and stability. Although the most active catalyst identified in this run shows slightly lower stability, it still surpasses the reference FeN_4_ catalyst in both metrics.

**Figure 3:**
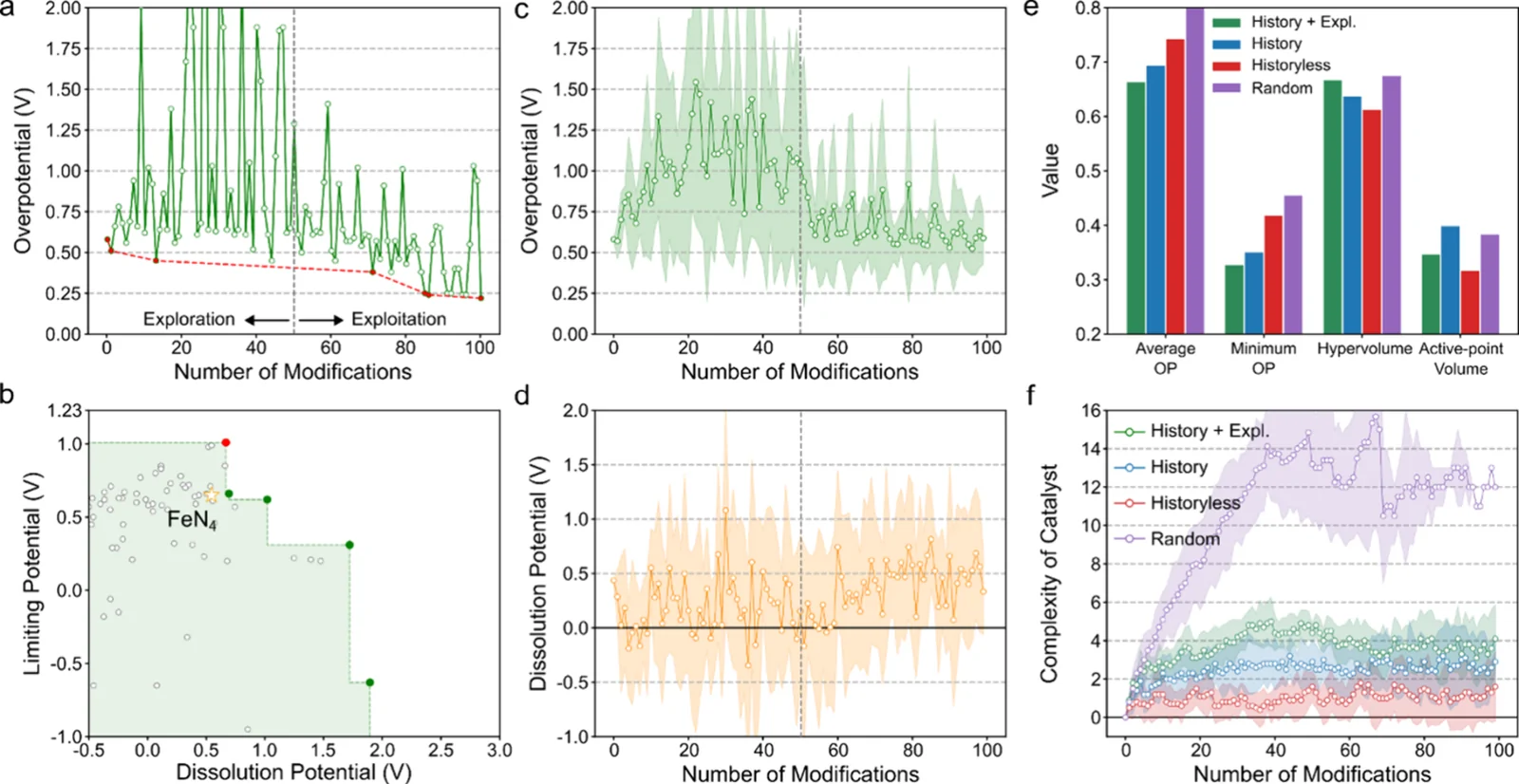
Performance and behavior of the MAESTRO framework across different strategies. (a) Representative example of the ORR overpotential change during a design run, illustrating the transition from the exploration phase to the exploitation phase after the 50th modification. The red dashed line denotes the running minimum overpotential. (b) Corresponding Pareto front obtained from the same run, where the red point highlights the most active catalyst discovered. (c) Evolution of overpotential and (d) dissolution potential, averaged over 10 independent design runs using “*history + exploration*” strategy. (e) Comparison of overall design performance across different strategies. (f) Evolution of catalyst complexity during the design run for each strategy, where a higher value indicates a larger number of modifications relative to the initial SAC structure.

Figure 3c and 3d summarize the average results over 10 independent design runs. Both graphs reveal a clear trend, where the overpotential gradually decreases while the dissolution potential increases following the transition to the exploitation phase (Figure S9). These observations demonstrate that the MAESTRO framework can effectively identify highly active SACs while maintaining or even improving their electrochemical stability. The origin of this enhanced discovery performance lies in the expansion of the accessible design space through the exploration phase and in the in-context learning enabled by the design history (Figure 3e). Our strategy exhibits the best performance in terms of both the exploitation-stage average overpotential and the minimum overpotential. Notably, even the “*history”* strategy consistently outperforms the “*historyless*” approach in average overpotential, underscoring the importance of leveraging accumulated design history. Detailed results and statistics about design run, MLFF-optimization and design performances are summarized in Figures S10 and S11 and Tables S9–S14.

In contrast, the hypervolume and active-point volume metrics show comparable values across the different strategies. This behavior arises because these metrics can be disproportionately influenced by high stability values, which are generally easier to achieve than low overpotentials. Therefore, maintaining Pareto volume metrics comparable to other strategies while simultaneously achieving lower overpotentials provides strong evidence that the framework effectively mitigates the activity-stability trade-off. Finally, the moderate level of catalyst complexity, defined by the number of modifications applied to the initial structure, indicates that our strategy maintains a balanced search behavior (Figure 3f). The design agent neither diverges excessively into chemically unrealistic structures nor becomes trapped in a narrow, suboptimal region of the design space.

### 2.4 Impact of In-Context Learning

To elucidate the origin of the observed performance of the MAESTRO framework, we investigated how in-context learning from design history influences the design process. We collected the binding energies of 2,017 unique catalysts from a total 4,000 structures generated across four discovery strategies, where each strategy was evaluated over 10 independent design runs consisting of 100 modification steps each. We then analyzed the linear scaling relations among the binding energies of the three ORR intermediates, *OOH, *O and *OH. This analysis confirmed the presence of well-defined linear scaling relations between ΔG_*O_ and ΔG_*OH_, as well as between ΔG_*OOH_ and ΔG_*OH_ in the SAC system (Figure 4a). Furthermore, by deriving a volcano plot using ΔG_*OH_ as the descriptor, we identified a theoretical minimum overpotential of about 0.36 V (Figure 4b). This value represents the lowest overpotential achievable by uniformly strengthening or weakening the binding energies of all intermediates while remaining on the scaling relations, without selectively modulating individual intermediates (Figure S12).

**Figure 4:**
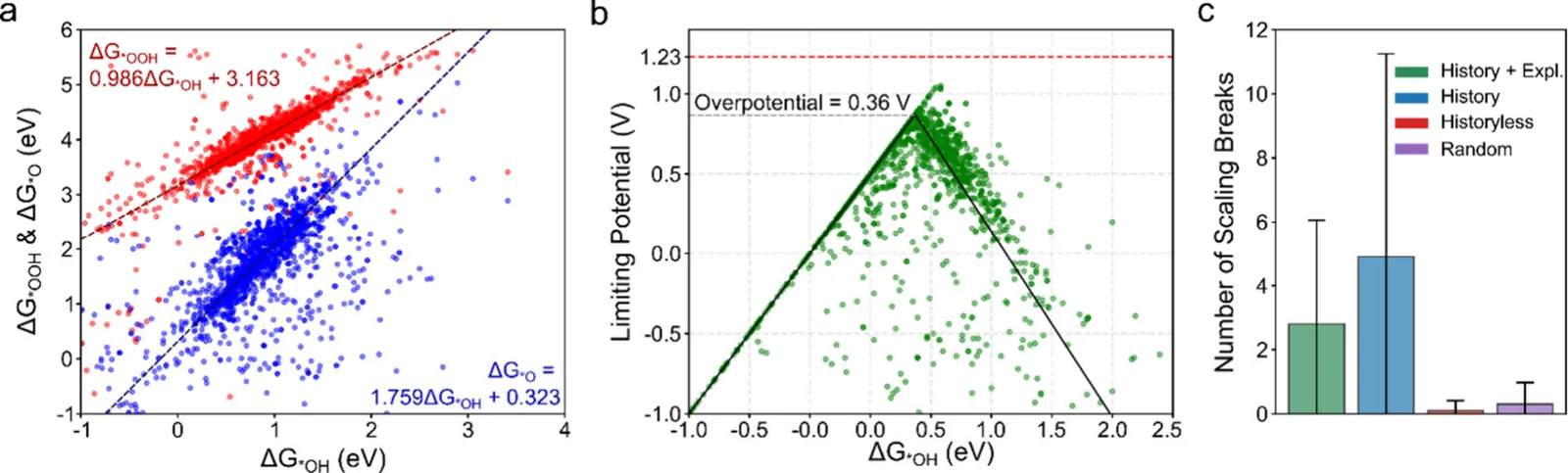
(a) Linear scaling relations among ΔG_*OH_, ΔG_*O_, and ΔG_*OOH_. (b) Corresponding volcano plot using ΔG_*OH_ as the activity descriptor. The red dashed line indicates the theoretical lower limit for overpotential 0.36 V imposed by the scaling relations. (c) Frequency of scaling relation violations observed during the design loop, averaged over 10 independent design runs for each strategy.

As we confirmed in Figure 2, the LLM possesses background chemical knowledge that enables it to propose modifications to control binding strengths as intended. Consistently, in Figure 3, the *historyless* strategy, which relies only on this background knowledge, exhibited a minimum overpotential exceeding 0.36 V. This observation indicates that strategies achieving overpotentials below 0.36 V must have been acquired through leveraging in-context learning from prior design steps. Specifically, such strategies learn to selectively tune the binding energy of specific intermediates, thereby breaking the inherent scaling relations rather than merely shifting all binding energies in tandem.^52^ This effect can be observed from the frequency of achieving overpotentials below the lower limit (Figure 4c). Across 10 design runs, both the *random* and *historyless* strategies achieved this value fewer than once on average, indicating that breaking the scaling relations in these cases occurs only sporadically by chance. In contrast, strategies with in-context learning from history broke these relations more than three times on average out of 100 modification steps. These results demonstrate that in-context learning from modification history plays a decisive role in overcoming fundamental scaling constraints (Figure S13), despite the higher token consumption compared to strategies without in-context learning (Figure S14).

### 2.5 Revealing Catalyst Design Principles

To elucidate the mechanism underlying the observed scaling relation breaking and the successful design of high-performance catalysts, we analyzed a representative designed catalyst in detail. Figure 5a shows an example of a catalyst achieving an overpotential of 0.31 V, surpassing the scaling-based lower limit of 0.36 V. During the design run, the design agent introduces surface oxygen functional groups (*COC or *COH). These surface oxygen species are observed to form H-bonds with the H atoms of *OH and *OOH intermediates, thereby stabilizing both species. As a result, only the ΔG_*O_ is selectively increased, leading to a break in the scaling relations between ΔG_*OH_ and ΔG_*O_. After inducing this scaling break, the agent retains the surface oxygen and subsequently explores first and second shell modifications, searching for configurations that further reduce the overpotential within the altered scaling landscape. Through this sequential process, the MAESTRO framework is able to design catalysts with overpotentials below 0.36 V. Importantly, this scaling break from selective H-bonds was confirmed not only at the MLFF but also through explicit DFT level. We performed DFT calculations on 11 catalysts selected from the FeN_4_ and CuN_4_-initiated design runs. Specifically, among the runs in which scaling relation break was observed, we selected only the single catalyst with the lowest MLFF-predicted overpotential from each run. Among these, six unique catalysts were confirmed by DFT to exhibit overpotentials below 0.36 V, and five of them featured surface oxygen species forming hydrogen bonds with ORR intermediates (Figure 5b). In addition to these minimum overpotential candidates, we performed DFT calculation for further catalysts whose MLFF-predicted overpotentials were below 0.36 V. The candidates for which the DFT-calculated overpotentials were substantially higher than 0.36 V were found to lack H-bonding and to exhibit disruption of local coordination shell induced by false positive activity predictions by the MLFF. This trend was also reflected in the Bader charge of the terminal H atom in the *OOH intermediate. The true positive candidates exhibited a mean H Bader charge of 0.605 e, whereas the false positive candidates showed a lower mean value of 0.577 e. The more positive charge on H in the true positive candidates is consistent with greater O–H bond polarization and stronger H-bonding stabilization of *OOH. Detailed structures and calculation results are provided in Figure S17 and Table S15.

**Figure 5:**
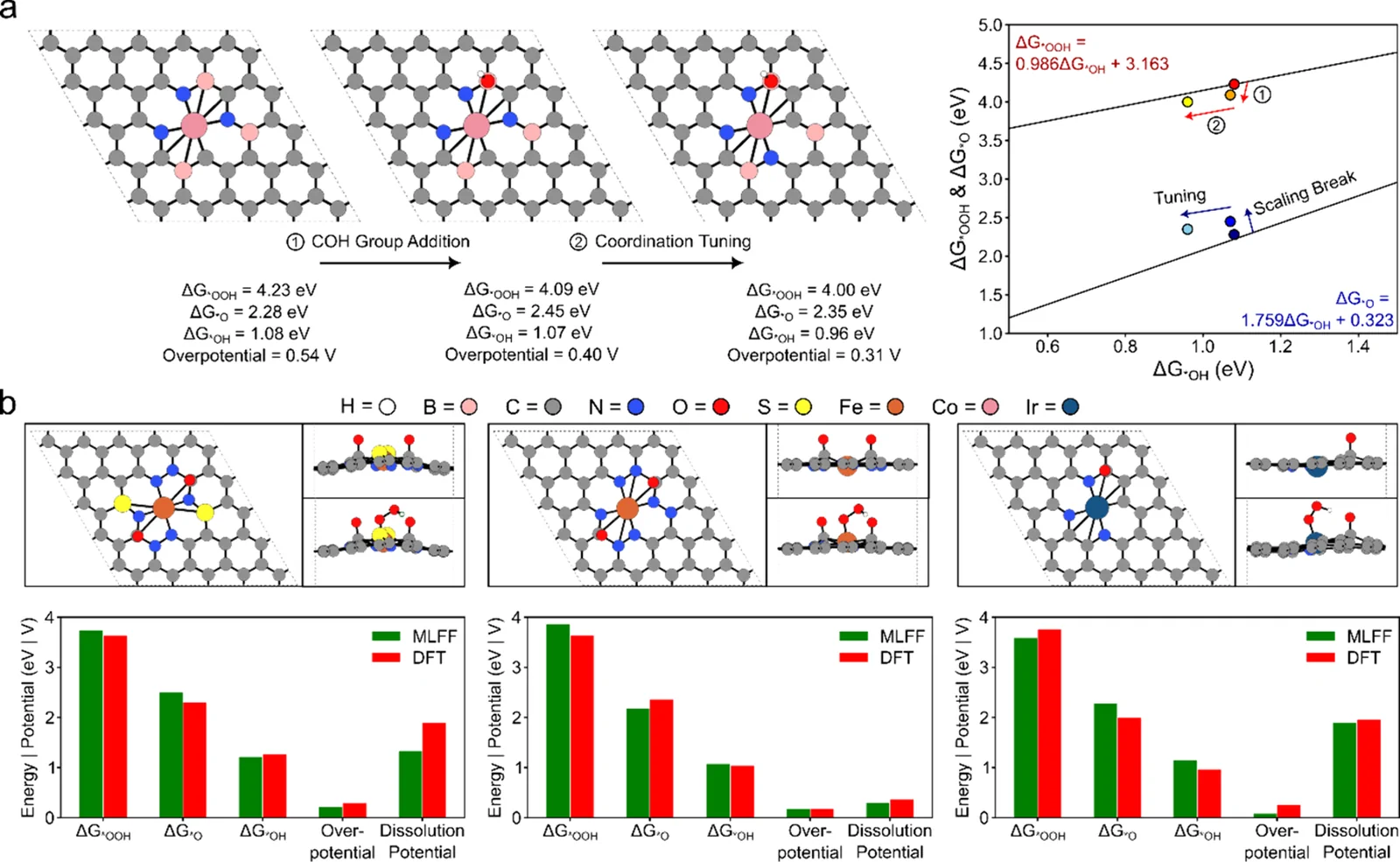
(a) Representative modification process to design promising catalysts by breaking scaling relations and lower limit of overpotential. Scaling break and overpotential optimization occur sequentially. (b) Representation of one of high-performance catalyst structures discovered during the design runs, together with a comparison of MLFF-predicted and DFT-calculated performance metrics. Data include Gibbs free binding energies, overpotential (η) and dissolution potentials (*U*_*diss*_) for each candidate. Other examples can be found in Figures S15 and S16.

The stabilization of intermediates via selective H-bonding, the resulting scaling break and the associated enhancement in catalytic performance have been reported in multiple prior studies.^53–57^ Therefore, the framework does not introduce a fundamentally new design principle, and the possibility that such knowledge was already partially encoded in the pretrained LLM cannot be excluded. However, as shown in Figure 4c, the agents without in-context learning rarely identified scaling break behavior, indicating that latent background knowledge alone was insufficient for effective application to the SAC design problem. Through iterative feedback and in-context learning, MAESTRO enabled the agents to retrieve and operationalize this knowledge as a system-specific design principles.^58^ These results suggest that the framework can not only optimize catalysts but also help translate latent chemical knowledge into actionable design strategies and potentially uncover novel catalyst design principles not explicitly provided through human input.

## 3 Discussion

### 3.1 Model, Starting State, and Hyperparameter Tuning

We have demonstrated that the MAESTRO framework operates successfully using GPT-4.1 mini as the LLM and FeN_4_ as the initial catalyst. We further examined how the choice of LLM, the starting SAC structure and key hyperparameters, including the LLM temperature and the number of recent design histories retained in short-term memory, affect the overall performance. In addition, we evaluate the performance of Bayesian optimization (BO) as an LLM free algorithmic benchmark for exploring the accessible SAC modification space.

First, BO was performed under a setting similar to that of MAESTRO, using the same overall SAC modification types and targeting the optimization of ORR overpotential. Because the full modification space is too large to be exhaustively explored, the substitution and defect positions in the first and second shells were fixed, and up to two substitution and defects were allowed. All other modification settings, as well as the total number of design iterations, were kept consistent with the MAESTRO setting, with each BO loop conducted for 100 iterations. Across 6 independent BO optimization loops, one run identified a candidate exhibiting scaling break behavior through hydrogen bonding. On average, BO achieved a minimum overpotential of about 0.408 V, indicating that BO is also a viable strategy for exploring promising SAC candidates for ORR (Figure S18). However, because BO performs purely algorithmic search without providing chemically interpretable reasoning, MAESTRO is better suited for analyzing how candidate structures are optimized, how scaling break emerges and how LLM agents acquire design principles through iterative feedback.

Next, we examined the effect of the LLM model itself. When the LLM was replaced with GPT-5 mini and Gemini-3.1-flash-lite while all other conditions were kept identical, the agents exhibited slightly more detailed reasoning and feedback. However, the overall performance remained comparable to that obtained with GPT-4.1 mini. This observation indicates that other LLMs possesses background knowledge similar to that of GPT-4.1 mini and that, in both cases, the ability to overcome scaling relations must be acquired through in-context learning during the design loop rather than being directly encoded in the model (Figure S19a).

We also investigated the effect of the starting material by initializing design runs with SACs containing other central metal atoms. In all cases, the average minimum overpotential achieved fell below 0.36 V, the limit dictated by the volcano plot. Moreover, each design run broke the scaling relations more than twice on average, demonstrating that our strategy is robust with respect to the choice of the initial metal center (Figure S19b). Among the starting structures, the design run initiated from PtN_4_ exhibited the best overall performance, except for the average overpotential. This behavior can be attributed to the intrinsically high initial overpotential of PtN_4_, which allows for broader exploration to the energy landscape during the exploration phase.

Finally, we evaluated the sensitivity of the framework to the temperature settings by performing 10 design runs across a range from 0.2 to 1.8. The results indicate that, provided the temperature remains within a moderate range, the overall discovery performance is largely comparable to the default setting of 1.0. In contrast, extreme values, such as 0.2 or 1.8, lead to a noticeable performance degradation (Figure S20).

### 3.2 Limitations and Future Directions

The types of SAC modifications employed in this study focus on fine-tuning the local environment of the active site by element substitution, atom addition/removal, and the introduction of functional groups or ligands. Although this approach expands the accessible design space, catalysts identified through such localized modifications may pose challenges for experimental synthesis. In particular, experimentally reproducing an identical local atomic environment remains difficult in practice, even when the designed structures satisfy electrochemical stability criteria. As a result, direct experimental validation of some discovered catalysts may be nontrivial. In future studies, incorporating more explicit synthesizability metrics or predictive models for synthetic accessibility will be important for bridging the gap between computational design and experimental realization.

One possible route to address this limitation is to integrate recent LLM-based approaches for synthesis planning, synthesizability assessment, and experimental recipe optimization. For example, Ramos et al. demonstrated that LLMs can be used for Bayesian optimization directly in natural language space by representing catalyst synthesis and testing procedures as prompts, enabling efficient optimization of catalytic recipes without explicit descriptor engineering.^59^ In a complementary direction, Kim et al. proposed Materealize as a multiagent platform for end-to-end inorganic materials design and synthesis, combining structure generation, property prediction, synthesizability prediction, and synthesis planning within a unified framework.^60^ Although such LLM-based systems are not yet sufficiently reliable to provide fully validated synthetic protocols for arbitrary SAC structures, they may offer useful guidance on experimentally relevant design variables, including precursor selection, thermal treatment conditions, ligand introduction strategies, defect generation methods and postsynthetic functionalization. Therefore, coupling the present design framework with LLM-based synthesis planning could provide a practical direction for improving the experimental realizability of computationally discovered SAC candidates.

Furthermore, this study is limited to the discovery of ORR catalyst within the SAC system. This choice was made to establish a proof-of-concept for agent-based catalyst discovery, leveraging SACs as a relatively well-defined platform, where the active site is spatially confined and the structural complexity is lower than in extended surfaces. We acknowledge, however, M-N_4_ SAC cannot fully represent the diversity of catalyst space, and therefore the generalizability of the present framework should not be overstated. Despite these simplifications, the present results demonstrate that the framework is capable of selectively modulating the binding energies of multiple intermediates while simultaneously managing activity, stability and structural complexity. Moreover, some of the reasoning evaluated in this work, such as the relationship between transition metal electronic structure and adsorbate binding strength, are broadly relevant to heterogeneous catalysis beyond SACs. Therefore, although the specific modification space is system-dependent, the overall strategy of combining LLM-guided reasoning with iterative feedback may be transferable to other catalyst systems. Future applications may include more complex systems, such as dual atom catalysts (DACs) or reactions like CO_2_ reduction, where the independent control of intermediate energies is even more critical.^61^

## 4 Conclusions

In this work, we proposed the MAESTRO framework and demonstrated that iterative interactions among specialized agents within an optimization loop can progressively enhance both catalytic activity and stability. Notably, the catalysts identified by the framework break the theoretical lower limit of ORR overpotential imposed by the conventional scaling relations. These findings indicate that the accumulation of design history, combined with agent-driven reasoning and self-reflection, can reveal new physical principles not explicitly encoded in the LLM’s background knowledge. Overall, our results suggest that the MAESTRO framework serves not only as an effective catalyst optimization tool but also as a means of autonomously generating novel chemical insights, significantly reducing the need for human intervention in the discovery of next-generation catalysts.

## 5 Methods

### 5.1 Large Language Models

A large language model (LLM) is a transformer-based autoregressive generative model trained on large-scale text corpora.^62^ By learning statistical and semantic patterns in language, an LLM is capable of performing complex reasoning, planning and decision-making tasks that resemble human cognitive processes. In this study, unless otherwise specified, all LLM-based agents were implemented using OpenAI’s GPT-4.1 mini model with default temperature and top-p settings. The detailed personas and system prompt templates assigned to each agent are provided in Supplementary Note A.

### 5.2 Machine Learning Force Field

Machine learning force field (MLFF) models serve as surrogate models for density functional theory (DFT) calculations. They are trained on large DFT data set to predict energy and atomic forces based on the geometry and stoichiometry of materials.^47^ These models typically interpret materials as graphs, where atoms are treated as nodes characterized by elemental features and interatomic bonds are encoded as edges containing geometric information. In this study, we employed the Universal Models for Atoms (UMA) as the MLFF.^49^ UMA is based on eSEN,^63^ an equivariant graph neural network that represents the atomic environment using spherical harmonic embedding and propagates information through multiple message passing layers. UMA is trained as a general-purpose model across diverse material domains and incorporates a Mixture of Linear Expert (MoLE) architecture to enhance flexibility and inference efficiency.^64^ For geometry optimization and energy prediction of the SACs, we utilized the pretrained UMA model corresponding to the ‘OC20’ domain,^65^ which is specifically weighted toward heterogeneous catalyst systems.

### 5.3 DFT Calculations

We performed DFT calculations using the Vienna Ab initio Simulation Package (VASP, version 5.4.4)^66,67^ to prevalidate MLFF performance and to evaluate SAC structures discovered by the catalyst design framework. To ensure consistency, we adopted DFT settings comparable to those used to generate the UMA training data. These settings were selected to provide a practical balance between computational cost and accuracy for the high-throughput evaluation of catalyst structures. The projected-augmented wave (PAW) pseudopotential method^68^ was employed in conjunction with the generalized gradient approximation revised Perdew–Burke–Ernzerhof (GGA-RPBE) exchange-correlation functional.^69^ All structures were fully relaxed until the total energy and atomic forces converged to within 10^–4^ eV and 0.05 eV/Å, for forces, respectively. A plane wave kinetic energy cutoff of 350 eV was applied. Monkhorst–Pack k-point mesh^70^ was configured as (3 × 3 × 1).

### 5.4 Gibbs Free Energy, Overpotential, and Dissolution Potential

Binding Gibbs free energies were evaluated using the computational hydrogen electrode (CHE) approach,^71^ in which the chemical potential of a proton–electron pair (H^+^ + e^–^) is referenced to 1/2H_2_ (g) at 0 V_RHE_ under standard conditions. Based on this framework, the Gibbs free energies for the adsorption of *O, *OH and *OOH intermediates were computed as follows:

$$\Delta G_{O*}=E_{O*}-E_{\mathrm{slab}}-E_{H_{2}O}+E_{H_{2}}+\Delta \left(\mathrm{ZPE+}\int C_{p}\mathrm{dT}-\mathrm{TS}\right)$$

$$\Delta G_{\mathrm{OH}*}=E_{\mathrm{OH}*}-E_{\mathrm{slab}}-E_{H_{2}O}+\frac{1}{2}E_{H_{2}}+\Delta \left(\mathrm{ZPE}+\int C_{p}\mathrm{dT}-\mathrm{TS}\right)$$

$$\Delta G_{\mathrm{OOH}*}=E_{\mathrm{OOH}*}-E_{\mathrm{slab}}+2E_{H_{2}O}+\frac{3}{2}E_{H_{2}}+\Delta \left(\mathrm{ZEP}+\int C_{p}\mathrm{dT}-\mathrm{TS}\right)$$

Here, E_*O_, E_*OH_, and E_*OOH_ denote the DFT total energies of the surface with the corresponding adsorbed intermediates, while *E*_H_2__ and *E*_H_2_O_ are DFT energies of gas phase H_2_ and H_2_O molecules, respectively. Zero-point energies (ZPE) corrections, enthalpic contribution (∫C_p_dT), and entropic contribution terms (TS) were calculated using the Harmonic oscillator approximation for adsorbed species (*O, *OH, *OOH) and the ideal gas approximations for gas molecules (H_2_, H_2_O), as implemented in Atomic Simulation Environment (ASE).^72^ The correction values are given in Table S16.

The theoretical overpotentials of ORR (η^ORR^) were determined from Gibbs free energy changes of the four elementary proton–electron transfer steps in the associative ORR pathway:

$$\Delta G_{1}=\Delta G_{*\mathrm{OOH}}-\Delta G_{O_{2}}+eU$$

$$\Delta G_{2}=\Delta G_{O*}-\Delta G_{*\mathrm{OOH}}+eU$$

$$\Delta G_{3}=\Delta G_{*\mathrm{OH}}-\Delta G_{*O}+eU$$

$$\Delta G_{4}=\Delta G_{H_{2}O}-\Delta G_{*\mathrm{OH}}+eU$$

The limiting potential (*U*_*L*_) is defined as the maximum potential at which all reaction steps become thermodynamically favorable (Δ*G*_*i*_(*U*_*L*_) ≤ 0 eV). The overpotential is the difference between the standard equilibrium potential for ORR (1.23 V) and the limiting potential, calculated as follows:

$$U_{L}=-\max\left[\Delta G_{1},\Delta G_{2},\Delta G_{3},\Delta G_{4}\right]/e$$

$$\eta^{ORR}=1.23-U_{L}$$

The dissolution potential of the SAC (*U*_*d*_) is an electrochemical stability, describing the tendency of the metal center to dissolve under ORR conditions. It is calculated as the difference between the binding energy of the metal atom to the carbon support (*E*_*b*_) and the standard dissolution potential of the corresponding bulk metal (*U*_*0*_) in aqueous solution (pH = 0), as follows:

$$E_{b}=E_{SAC‐M}-E_{SAC}-\mu_{M}$$

$$U_{d}=U_{0}-E_{b}/\left(e{\times N}_{e}\right)$$

where *E*_SAC‑M_ and *E*_SAC_ are the DFT total energies of the carbon support with and without the metal atom, respectively. μ_M_ is the chemical potential of the metal atom, *e* is elementary charge and *N*_*e*_ is the number of electrons involved in the metal dissolution process.

## Supplementary Material



## Data Availability

The code developed in this work and relevant information can be found in GitHub (https://github.com/ahrehd0506/Catalyst-Design-Agent).

## Notes

Details of prompt template, details of prevalidation for MLFF and LLM, example of exploration summary report and reasoning of the agents, additional metrics for design framework, metrics for strategies with various starting materials, parameter and LLM model (PDF)
