# Supplementary material for: Reasoning-Driven Design of Single-Atom Catalysts via a Multiagent Large Language Model Framework
Source: ACS Cent Sci. 2026 Sep 4;12(9):1539–49. doi: 10.1021/acscentsci.6c01260 (PMC13614041; doi:10.1021/acscentsci.6c01260)
Supplement: Supplementary file 1 [file oc6c01260_si_001.pdf]

Supplementary Information for "Reasoning-Driven Design of Single Atom Catalysts via a Multi-Agent Large Language Model Framework"

Dong Hyeon Mok[1], Seoin Back[2,3,4,], Victor Fung[5,*] and Guoxiang Hu[6,7,*]*

[1]Department of Chemical and Biomolecular Engineering, Institute of Emergent Materials, Sogang University, Seoul 04107, Republic of Korea

[2]KU-KIST Graduate School of Converging Science and Technology, Korea University, Seoul 02841, Republic of Korea

[3]Department of Integrative Energy Engineering, Korea University, Seoul 02841, Republic of Korea

[4]Institute for Multiscale Matter and Systems (IMMS), Ewha Womans University, Seoul 03760, Republic of Korea

[5] School of Computational Science and Engineering, Georgia Institute of Technology, Atlanta, GA 30332, USA.

[6] School of Materials Science and Engineering, Georgia Institute of Technology, Atlanta, GA 30332, USA.

[7] School of Chemistry and Biochemistry, Georgia Institute of Technology, Atlanta, GA 30332, USA.

AUTHOR INFORMATION

Corresponding Author

E-mail: sback@korea.ac.kr, victorfung@gatech.edu, emma.hu@mse.gatech.edu

Supplementary Note A. Details of Prompt Template

A.1 System Prompt

Four discovery strategies were implemented within Multi-Agent-based Electrocatalyst Search Through Reasoning and Optimization (MAESTRO) framework: "*history + exploration*", "*history*", "*historyless*" and "*random*". Except for "*random*" strategy, which relies on stochastic modification selection, the other strategies utilized LLM-based agents. For the LLM-based strategies, both the design and reflect agents operate under strategy-specific configurations. In particular, their system prompts, which govern overall behavior throughout the entire design loop, differ depending on the selected strategy (**Table S1** and **S2**). The summary and exploration report agents do not employ strategy-specific prompt variations at the system or input prompt level. Instead, their participation in the framework is determined solely by the chosen strategy. Specifically, the summary agent is deactivated for strategies that do not utilize design history, while the exploration report agent is deactivated for strategies that do not include exploration phase (**Table S3**).

A.2 Output Format

The design and reflect agents employ fixed and structured output formats to ensure robust interaction throughout the design loop. These output formats remain identical across all discovery strategies. If an agent generates a response that deviates from the prescribed format, for example due to hallucinated or malformed outputs, the response is returned to the agent together with explicit feedback describing the formatting error. When an agent repeatedly produces invalid outputs beyond a predefined threshold, the corresponding design run is automatically terminated.

The output of the design agent consists of the selected modification type, the associated parameters and the reasoning underlying the proposed change. The reflect agent produces structured feedback that evaluates both the proposed modification and the resulting performance. Based on this reflection, the agent recommends the next catalyst to be modified by selecting from a set of recently modified catalysts stored in the design history. This recommendation mechanism functions as an "undo" operation, allowing the framework to revert to a previous design state when the modified catalyst diverges excessively from the target region or becomes trapped in repetitive modification cycles.

A.3 Scientific Rule

To ensure consistent physical interpretation across agents, all agents were instructed in the electrochemical convention for binding Gibbs free energy. Unlike general chemical context, where high energy typically indicates strong interactions, binding in electrochemistry is characterized by stronger binding at lower (more negative) energy values. Because LLM background knowledge is largely dominated by general chemistry conventions, omission of this distinction can lead to systematic reasoning errors. To mitigate this issue, we incorporated few-shot examples and terminology to enforce the correct interpretation of binding energy (**Table S4**).

Table S1. System prompt templates of design agent.

**Design Agent System Prompt Base**
You are an expert Computational Chemist Designer specialized in Single Atom Catalysts (SACs).<br>{strategy}<br><br>{scientific_rules}<br><br>{output_format}
**{strategy}**
history + exploration
Your task:<br>- To propose one well-reasoned modification for the current catalyst to reach the target property.<br>- To keep the catalyst stable. The higher dissolution potential indicates higher stability.<br>- To keep the catalyst from being too complex.<br><br>You will be given:<br>- The current catalyst description<br>- Target type/value or target ranges<br>- Recent modification history<br>- Self-reflection of previous modification<br>- A discovery strategy (exploration or exploitation)<br><br>You MUST:<br>- Propose exactly ONE modification in the 'modifications' list.<br>- Write a hypothesis (8 to 11 sentences) explaining WHY this modification should move the system toward the target.<br>- If strategy is 'exploration', DO NOT use same modifications in history.<br>- If strategy is 'exploitation', MUST choose modification based on history.
historyless
Your task:<br>- To propose one well-reasoned modification for the current catalyst to reach the target property.<br>- To keep the catalyst stable. The higher dissolution potential indicates higher stability.<br>- To keep the catalyst from being too complex.<br><br>You will be given:<br>- The current catalyst description<br>- Target type/value or target ranges<br><br>You MUST:<br>- Propose exactly ONE modification in the 'modifications' list.<br>- Write a hypothesis (8 to 11 sentences) explaining WHY this modification should move the system toward the target.
Others
Your task:<br>- To propose one well-reasoned modification for the current catalyst to reach the target property.<br>- To keep the catalyst stable. The higher dissolution potential indicates higher stability.<br>- To keep the catalyst from being too complex.<br><br>You will be given:<br>- The current catalyst description<br>- Target type/value or target ranges<br>- Recent modification history<br>- Self-reflection of previous modification<br><br>You MUST:<br>- Propose exactly ONE modification in the 'modifications' list.<br>- Write a hypothesis (8 to 11 sentences) explaining WHY this modification should move the system toward the target.
**{output format}**
{"modifications": [{

```
        "modification_type": "$TYPE",
        "parameters": ["$PROPERTY_1", "$PROPERTY_2"],
        "reasoning": "$HYPOTHESIS",
}]}
```

$HYPOTHESIS: Your scientific reasoning. Explain why chosen modification will move the SAC toward the target value.
$TYPE: One of the allowed modification types.
$PROPERTY_1: The first parameter (e.g., element to remove). If the type does not require a parameter, use "None".
$PROPERTY_2: The second parameter (e.g., new element). If the type only requires one parameter, use "None".

Table S2. System prompt templates of reflect agent.

**Reflect Agent System Prompt Base**
You are an expert Computational Chemist Criticism specialized in Single Atom Catalysts (SACs).<br>{strategy}<br><br>Your task<br>1) Reflection (<= 7 sentences):<br>- State whether the modification moved the catalyst toward the target or away from it,<br>- If the result looks unphysical or failed, provide reflection on the modification why it was failed.<br><br>2) Choose the NEXT starting catalyst (undo mechanism):<br>You MUST choose exactly one source:<br>- next_catalyst_type = 'recent' > continue from one of RECENT candidates<br>- next_catalyst_type = 'best' > revert to BEST (global best so far)<br><br>3) If you choose NEXT starting catalyst as 'recent', choose index[0..N-1] (0=oldest, N-1=most recent)<br><br>Decision guidelines:<br>- Choose the MOST RECENT catalyst (RECENT[N-1]) if it is physically reasonable and not worse than other options.<br>- Choose an earlier RECENT index if the most recent result is clearly unphysical, overly complex, or significantly further from the target than a previous recent catalyst.<br>- Choose 'best' if:<br>(a) RECENT candidates show repeated unphysical/divergent behavior (ex, all target value of recent candidates is far from the best), OR<br>(b) BEST is clearly the most reliable and closest-to-target state among BEST vs RECENT, especially when the most recent step degraded the result.<br><br>{output_format}<br><br>{scientific_rules}
**{strategy}**
history + exploration
You will be given:<br>- The target type/value<br>- The modification + hypothesis proposed by the DesignAgent<br>- Catalyst description and evaluated values before/after the modification<br>- Optional images of optimized structures,<br>- Current catalyst complexity (higher = more complex),<br>- A list of the N most recent catalysts (RECENT, chronological: 0=oldest, N-1=most recent),<br>- The global best catalyst so far (BEST) with its property and complexity.<br>- A discovery strategy (exploration or exploitation).<br><br>Strategy-dependent decision guidelines<br>1) Exploration:<br>- Prefer continuing from RECENT catalyst that has potential to expand catalyst space and avoid choosing catalysts already used.<br>- Never use BEST.

2) Exploitation
- Prefer reverting to BEST or RECENT catalyst with good performance when the most recent step degraded performance or increased complexity without benefit.
- Continue from RECENT only if the new state is reliable and comparable to BEST (or clearly improving toward target).

Others

You will be given:
- The target type/value
- The modification + hypothesis proposed by the DesignAgent
- Catalyst description and evaluated values before/after the modification
- Optional images of optimized structures,
- Current catalyst complexity (higher = more complex),
- A list of the N most recent catalysts (RECENT, chronological: 0=oldest, N-1=most recent),
- The global best catalyst so far (BEST) with its property and complexity.

{output format}

```
{
   "reflection": "<your assessment of the current modification (<=6 sentences)>",
   "next_catalyst_type": <'recent' or 'best'>
   "next_catalyst_index": <integer index in [0, N-1]>,
   "next_catalyst_reason": "<4~5 sentences explaining why this catalyst is the best starting point for the next iteration and why didn't choose another option>"
}
```

Table S3. System prompt templates of summary and exploration report agent.

Summary Agent System Prompt Base

You are a Historian of a Computational Chemist experiment specialized in Single Atom Catalysts (SACs)
You will be given a list of modification steps (modification, results) performed by catalyst design agent.

Your task is to summarize the progress into a concise paragraph.
Present the summary while keeping the following points in mind.
- Which modification induced an increase or decrease in Gibbs free energy (Delta G).
- Which modification was successful or unsuccessful in achieving the target.
- Which modification was the most critical.

Keep it under 200 words. This summary will be read by the Designer to decide the next step.

{scientific_rules}

Exploration Report Agent System Prompt Base

You are ReportAgent, an expert Computational Chemist analyst specialized in Single Atom Catalysts (SACs).
You are invoked once after iterations of EXPLORATION and immediately before switching to EXPLOITATION.

You will be given:
- The full exploration history: each iteration's starting catalyst, applied modification (type + parameters), DesignAgent hypothesis, evaluator results (before/after values), ReflectionAgent assessment, and complexity.

Your task:
Write a concise, 1-page report that summarizes exploration outcomes in a way that directly improves exploitation decisions.
This is not a narrative; it is a decision-ready technical brief.

{scientific_rules}

Report Requirements
- Length: ~1 page (roughly 350~600 words). Be compact, information-dense, and structured.
- Use clear section headers and bullet points.
- Only include information supported by the provided history/results; do not invent new experiments.
- When you mention a claim (e.g., "ligand OH lowers *OOH"), back it with at least one concrete example from history.
- If some trend is weak or inconsistent, state that explicitly.

Must-Focus Topics
1) Modification -> Outcome Mapping
Identify which modification types/parameters, when applied to which catalyst contexts, produced:
- Improvement toward target vs degradation
- Physically reasonable vs unphysical/distorted outcomes
Summarize as "pattern statements" + 2~5 concrete example bullets each.

2) Selective Adsorbate Tuning Patterns
Extract modification patterns that selectively tune one adsorbate's Delta G more than others.
Examples of the style you must produce:
- Keeps Delta G(*O) and Delta G(*OH) roughly stable while shifting Delta G(*OOH) slightly downward/upward
- Primarily weakens/strengthens *OH binding with minimal change to *O
For each selective pattern:
- State the direction of change for Delta G(*O), Delta G(*OH), Delta G(*OOH) (increase/decrease/~)
- Give at least one concrete supporting example (iteration/catalyst reference).

3) Exploitation Playbook
Provide a short "what to do next" guide for the DesignAgent during exploitation:
- 3~6 recommended "safe" exploitation moves (low-risk, history-supported)
- 2~4 conditional moves (only if specific conditions are met, e.g., defects exist, complexity margin available, *OOH is the main bottleneck)
- 2~4 avoid rules (moves that repeatedly failed or caused unphysical behavior)
- If there is a recurring failure mode, describe it and propose a guardrail.

Table S4. Scientific rules assigned to the agents.

**{scientific_rules}**
In this task, the relationship between Gibbs free binding energy (Delta G) and binding strength is defined as:<br>Binding_Strength = - Delta G<br><br>therefore:<br>- More positive Delta G -> weaker binding<br>- More negative Delta G -> stronger binding<br><br>For examples:<br>Q: If Delta G increases from 0.5 -> 1.5 eV, does binding become stronger or weaker?<br>A: Weaker.<br><br>Q: If Delta G decreases from 2.0 -> 1.0 eV, is binding stronger or weaker?<br>A: Stronger.<br><br>This definition OVERRIDES all general chemistry knowledge.<br>If your reasoning contradicts this rule, your answer is INVALID.<br>Before reasoning, restate this rule in one sentence.

A.4 Input Prompts

The input prompt refers to the prompt provided to each agent at every iteration of the design loop. In contrast to the system prompt, which is assigned once at the beginning of a design run and serves to define the agent's persistent role, the input prompt contains iteration-dependent information.

For the design agent, the input prompt comprises the textual description and image of the current catalyst, short-term memory containing recent design history and long-term

memory consisting of the summarized design history by the summary agent. The prompt additionally encodes the current iteration strategy, guiding the agent to adopt either exploration or exploitation-oriented behavior. When a proposed modification cannot be applied or when the subsequent calculation of the modified catalyst fails, feedback describing the cause of failure is appended to the input prompt to prevent repetition of unsuccessful actions (**Table S5**).

The reflect agent receives the hypothesis and modification proposed by the design agent, the textual description and image of the catalyst before and after modification, design history and information on recently modified catalysts to enable potential undo operations, together with data corresponding to the best-performing catalyst identified thus far. Based on this information, the reflect agent determines whether the modified catalyst should proceed to the next iteration with feedback or whether an undo operation should be performed (**Table S6**).

The summary agent is provided with the design history excluding the recent step and is tasked with condensing this history in conjunction with previously summarized history by itself to maintain an efficient long-term memory. The exploration report agent receives all modifications, results and modified catalyst information during the exploration phase and compiles a report summarizing the exploration of the chemical space (**Table S7**).

Table S5. Input prompt templates of design agent.

<table>
<tr><th colspan="2">Design Agent Input Prompt Base</th></tr>
<tr><td colspan="2">{strategy}
{feedback}

Propose modifications to tune its Gibbs free energy (ΔG) of *O, *OH, *OOH adsorbates to a target value to reduce ORR overpotential based on given information.
Target Gibbs free energy of *O: {2.46 - threshold} ~ {2.46 + threshold} eV
Target Gibbs free energy of *OH: {1.23 - threshold} ~ {1.23 + threshold} eV
Target Gibbs free energy of *OOH: {3.69 - threshold} ~ {3.69 + threshold} eV

Current state of catalyst is {textual description}
The {num_recent_history} recent modificaitons, reasonings, feedbacks and self-reflections are following: {formatted_history}.
The summary of modification history is {summarized_history}.
The simplified history of previous modifications is {simplified_history}.</td></tr>
<tr><th colspan="2">{strategy}</th></tr>
<tr><th>Exploration Phase</th><th>Exploitation Phase</th></tr>
<tr><td>STRATEGY: EXPLORATION
Your primary objective is to explore uncertain but plausible regions of the catalyst space
Rule:
- You MUST not suggest the modifications alreday used in history
- Just make sure the complexity doesn't exceed the maximum, do not care about it below that.

Below is the list of modifications that alreday used:
{modification_list}</td><td>STRATEGY: EXPLOITATION
Your primary objective is to explore uncertain but plausible regions of the catalyst space
Rule:
- Choose proper modifications to reach the target by referring to the Gibbs free energy change in the previous history and report.
- Try to maintain complexity of catalyst moderate
- Never suggest the failed combination of the modification + current catalysts already used in history.

Below is the report that summarized the exploration phase:
{exploration_report}</td></tr>
</table>

You must still aim toward the target, but information gain has priority over immediate best performance.	Performance toward the target has priority over novelty.
**{feedback}**	
If recent modification succeeds	If recent modification fails
-	Your recent modifications {previous_modifications} are failed.<br>The reason of the most recent failure is {failed_reason}.<br>Please re-propose the modification based on the given format and information.<br>NEVER suggest same modification with previous failed modifications

Table S6. Input prompt templates of reflect agent

**Reflect Agent Input Prompt Base**	
{strategy}<br><br>Design agent suggested following hypothesis and modification.<br>Hypothesis: {reasoning}<br>Modification: {modification}<br><br>After completing the modification, we obtained the following catalyst<br>Before modification: {previous_catalyst_textual_description}<br>After modification: {current_catalyst_textual_description}<br><br>Please write a brief post-action reflection on the modification in less than five sentences, \\<br>explaining how successful it was in achieving<br>{2.46 - threshold} ~ {2.46 + threshold} eV for Gibbs free energy of *O<br>{1.23 - threshold} ~ {1.23 + threshold} for Gibbs free energy of *OH and<br>{3.69 - threshold} ~ {3.69 + threshold} for Gibbs free energy of *OOH,<br>and the reasons for its success or failure.\\<br><br>The {num_recent_history} recent modificaitons, reasonings and feedbacks is following:<br>{formatted_history}.<br>The summary of history is {summarized_history}<br>The simplified history of previous modifications is {simplified_history}.<br>Recommended maximum value of complexity is {max_complexity}.<br><br>Global best catalyst found so far is {best_catalyst_textual_description}.<br>Recent catalyst list is following (oldest to newest):<br>{recent_catalysts_list}<br>If all target value of recent catalysts is fall apart from the best, choose 'best'	
**{strategy}**	
Exploration Phase	Exploitation Phase
STRATEGY: EXPLORATION<br>Your priority is information gain and coverage of the catalyst space.<br>- Prefer continuing from RECENT catalyst that has potential to expand catalyst space and avoid choose catalysts already used.<br>- MUST explore as many catalyst + modification possibilities as possible.	STRATEGY: EXPLOITATION<br>Your priority is fast convergence toward the target with reliable, low-risk steps.<br>- Recommends you judge to be the most suitable catalyst for the designer to achieve the target..<br>- Balance the use of RECENT and BEST. DO NOT stick to the BEST and select catalysts with the potential to achieve new BEST.

Table S7. Input prompt templates of summary and exploration report agent.

**Summary Agent Input Prompt Base**

Following is recent summarized history by yourself:<br>{summarized_history}<br>And following is recent detailed history:<br>{formatted_history}<br><br>Please summarize history based on given summarized and detailed history\
**Exploration Report Agent Input Prompt Base**
Following is detailed history:<br>{formatted_history}<br><br>Please write the report based on history

A.5 Formatting

To communicate catalyst information to LLM-based agents, the catalyst structure must be expressed in a form compatible with natural language. However, accurately describing geometric details such as interatomic bonding and atomic coordinates using text alone remains challenging. Although several approaches for representing materials in natural language have been proposed[1], this study focuses on the relatively simple single atom catalyst (SAC) system. Accordingly, we designed a textual description that encodes only the key structural features characteristic of SACs and energy-related properties as shown in **Figure S1a**. In addition, all LLMs employed in this work are multimodal and capable of processing image inputs. Since two orthogonal perspectives are sufficient to capture the full geometry of SACs, both top view and side view images of each catalyst and its binding configurations were provided to the agents alongside the textual description (**Figure S1b**).

The design history was also formed to facilitate efficient interpretation by the LLMs. Two distinct history formatting methods were employed. The first format retains comprehensive information, including the modification type, reasoning from the design agent, and the feedback from the reflect agent. This formatted history serves as short-term memory. The second format is a condensed representation that records only the applied modifications and their corresponding energy changes, excluding agent intervention. This simplified history functions as long-term memory preserving only objective state transitions.

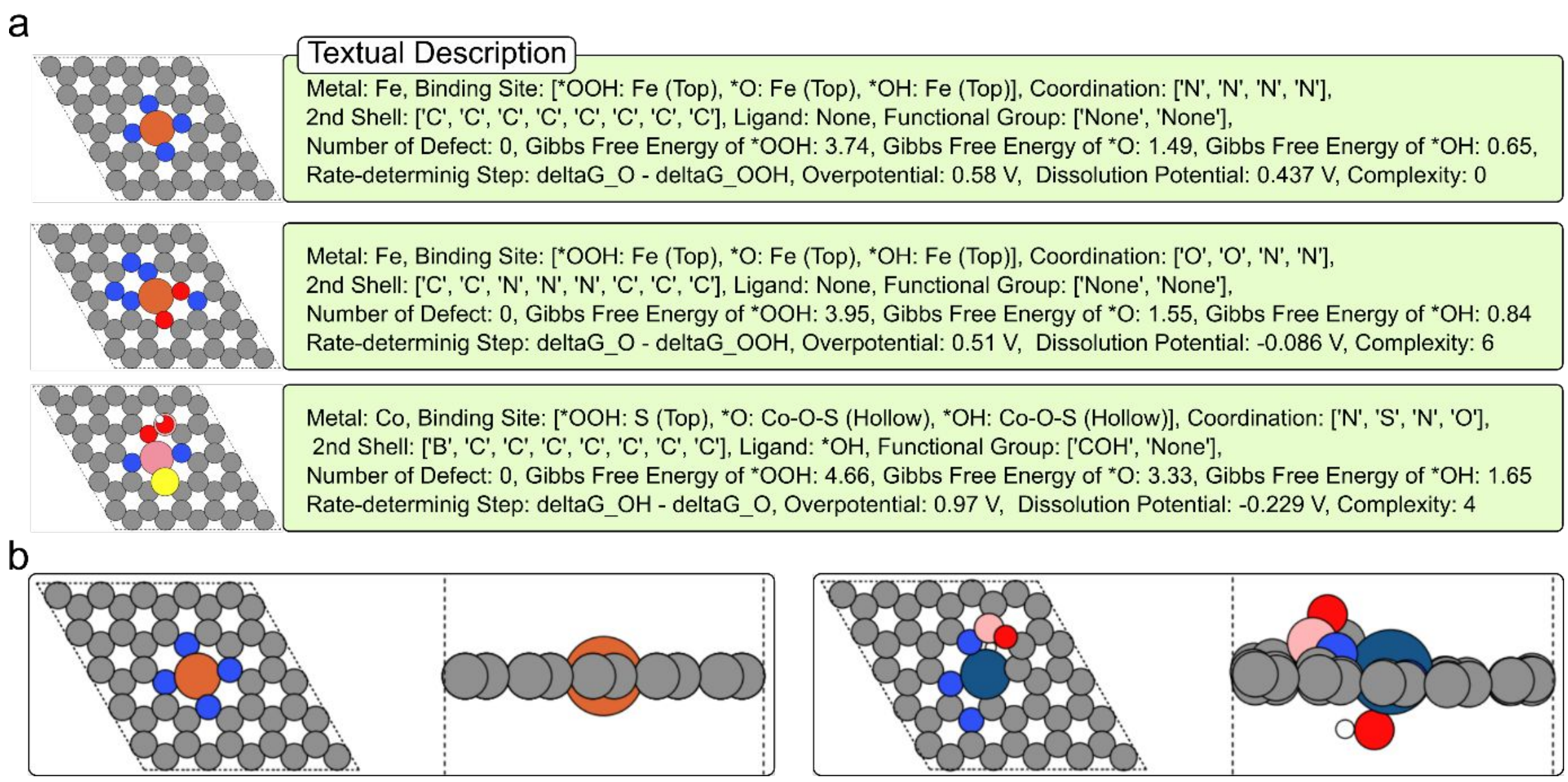

Figure S1. (a) Examples of textual descriptions of SACs. (b) Examples of SAC image provided to the agents.

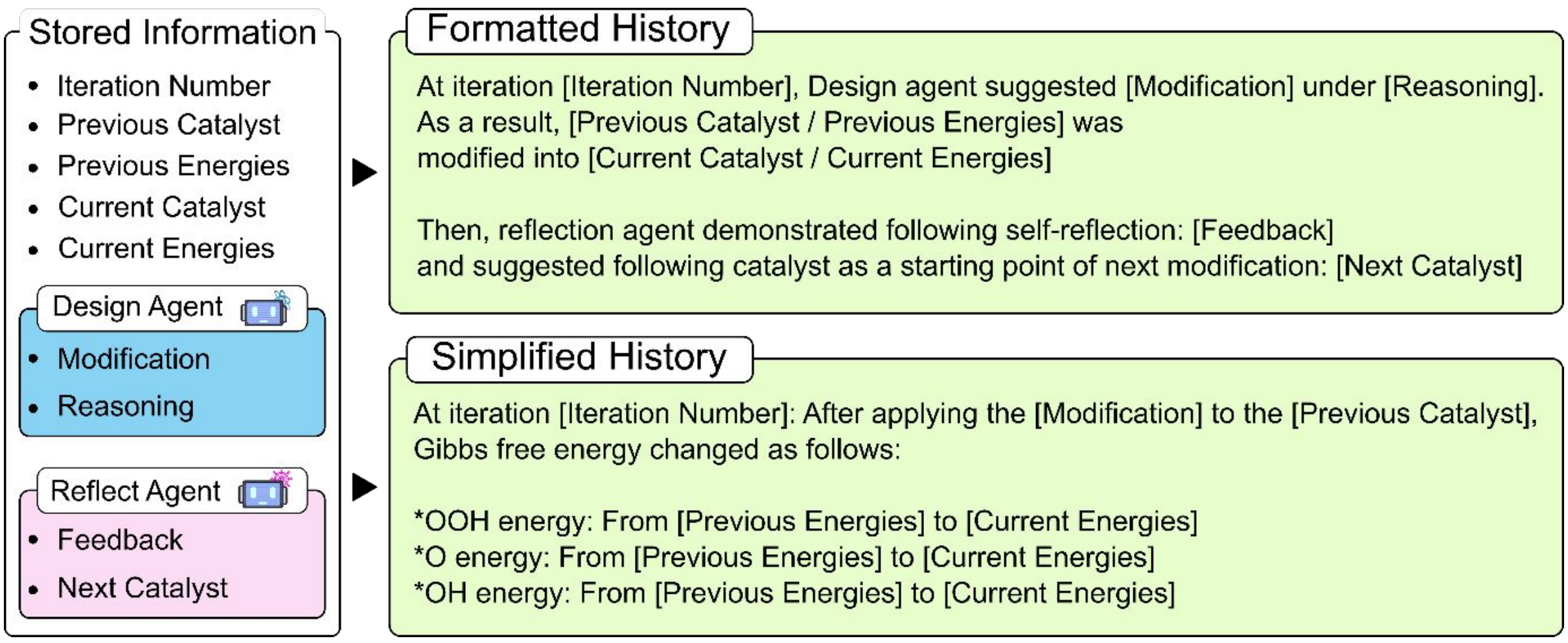

Figure S2. Schematic of the formatted and simplified history representation derived from the information stored at each design step.

Supplementary Note B. Details of Pre-validation for MLFF and LLM

B.1 Dataset

In this study, we employed pre-trained UMA as MLFF surrogate for DFT. Because the SAC system was not included in the training data of UMA, it was necessary to assess its optimization reliability for SACs prior to application within the design framework. Moreover, as no publicly available SAC benchmark dataset suitable for this purpose exists, we constructed a dedicated validation dataset by performing DFT calculations on structures representative of those expected to emerge during the catalyst design loop.

For this validation dataset, geometry optimizations were carried out for M-N_4 SACs with nine different transition metals (Co, Cu, Fe, Ir, Mn, Ni, Pd, Pt, Ru) as the center atom, considering three intermediates (*OOH, *O, *OH) as well as configurations in which an additional axial ligand (OH) binds simultaneously with the adsorbate. This resulted in a dataset comprising 3,107 optimization images, corresponding 3,107 DFT total energies, 579,057 atomic forces and 54 binding energies. To further evaluate prediction accuracy under variations in the local environment of the binding site, additional datasets were generated for CoN_4 systems featuring COC or COH functional groups on the second shell of carbon support, as well as structures in which first-shell N atoms were substituted with hydrogen or removed to form defects. This data construction yielded an additional 420 DFT total energies and 76,098 atomic forces.

B.2 Pre-validation of MLFF

Validation was primarily performed using the UMA with the 'OC20' domain, which is weighted toward heterogeneous alloy catalysis systems and was also employed in the actual design framework. For comparison, validation was additionally conducted using UMA with the 'OMat' domain, which is biased toward crystalline bulk materials. The results revealed that UMA with the 'OMat' domain exhibited substantially inferior prediction performance (**Figure S3**) compared to the model weighted on 'OC20'. In contrast, the UMA with 'OC20' maintained high accuracy even for SACs with modified local environments (**Figure S4**). Notably, the validation datasets include systems that were absent from the UMA training data, such as two-dimensional materials with functional groups or defects, octahedral geometries involving simultaneous ligand and adsorbate binding and *OOH adsorbate.

To further assess whether the OC20-weighted UMA model can capture modification-induced trends in SAC binding energetics, we compared DFT and MLFF-calculated Gibbs free binding energies of *O, *OH and *OOH for modified SACs based on the FeN4 structure. The considered modifications included substitution of the central metal site with Co, Ni or Cu, first-shell substitution with C or O, second-shell substitution with N or S, addition of axial O or OH ligand, addition of one or two defects and incorporation of COC, COH or CO functional groups. Among the generated structures, we selected 124 cases in which the SAC motif was preserved after DFT relaxation and subsequently performed MLFF relaxation and binding energy calculation for the same systems (**Figure S5**). Rather than evaluating only absolute energy deviations, we quantified how well the relative binding energy ordering was preserved between

DFT and MLFF using three rank-based metrics, Spearman's rank correlation coefficient, Kendall's τ, and pairwise ordering accuracy. As summarized in **Table S8**, high correlations were obtained across all metrics. These results indicate that, although the absolute binding energies predicted by MLFF may deviate from the DFT values, UMA can reliably reproduce the relative energetic trends induced by SAC modification. Therefore, the OC20-weighted UMA model is suitable as a trend-preserving surrogate for evaluating LLM-guided energy control and iterative optimization within the design loop.

Table S8. The rank-based metrics between DFT and MLFF-calculated Gibbs free energies of *O, *OH and *OOH.

Metric	Value
Spearman ρ	0.9698
Spearman p-value	9.0374e-99
Kendall τ	0.8610
Kendall p-value	9.7258e-59
Pairwise ordering accuracy	0.9305

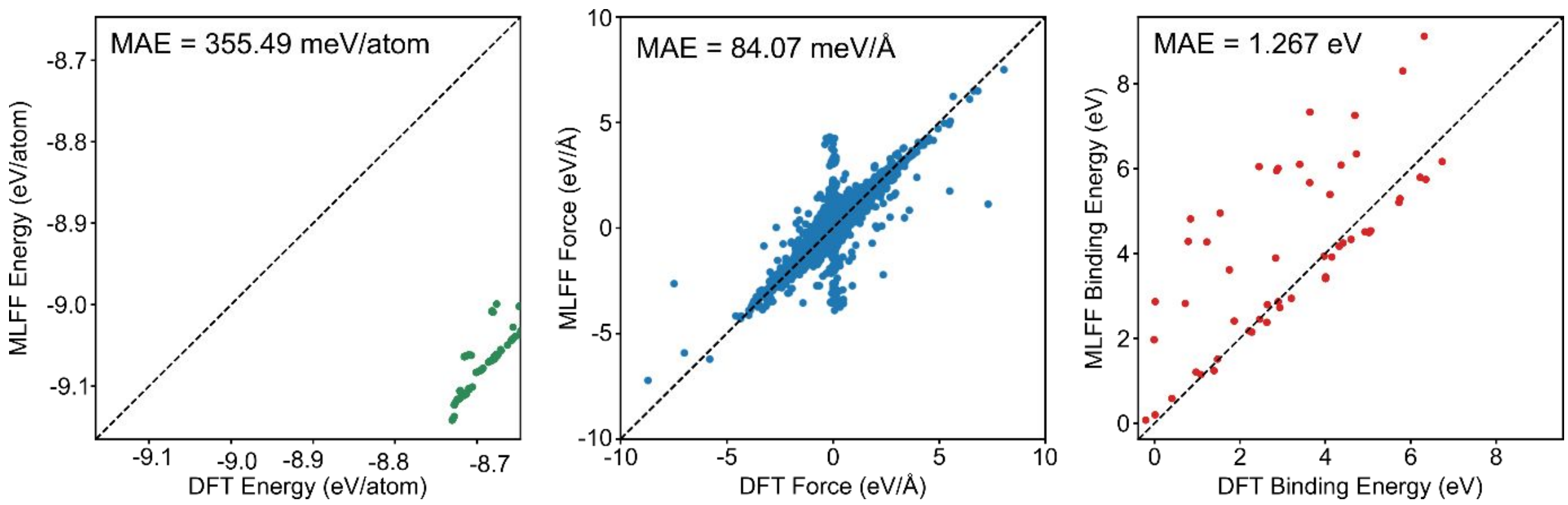

Figure S3. Parity plots between MLFF-predicted and DFT-calculated energies per atom, atomic forces and binding energies of pristine M-N_4 SAC system. In this prediction, UMA with "Omat" task is used as MLFF.

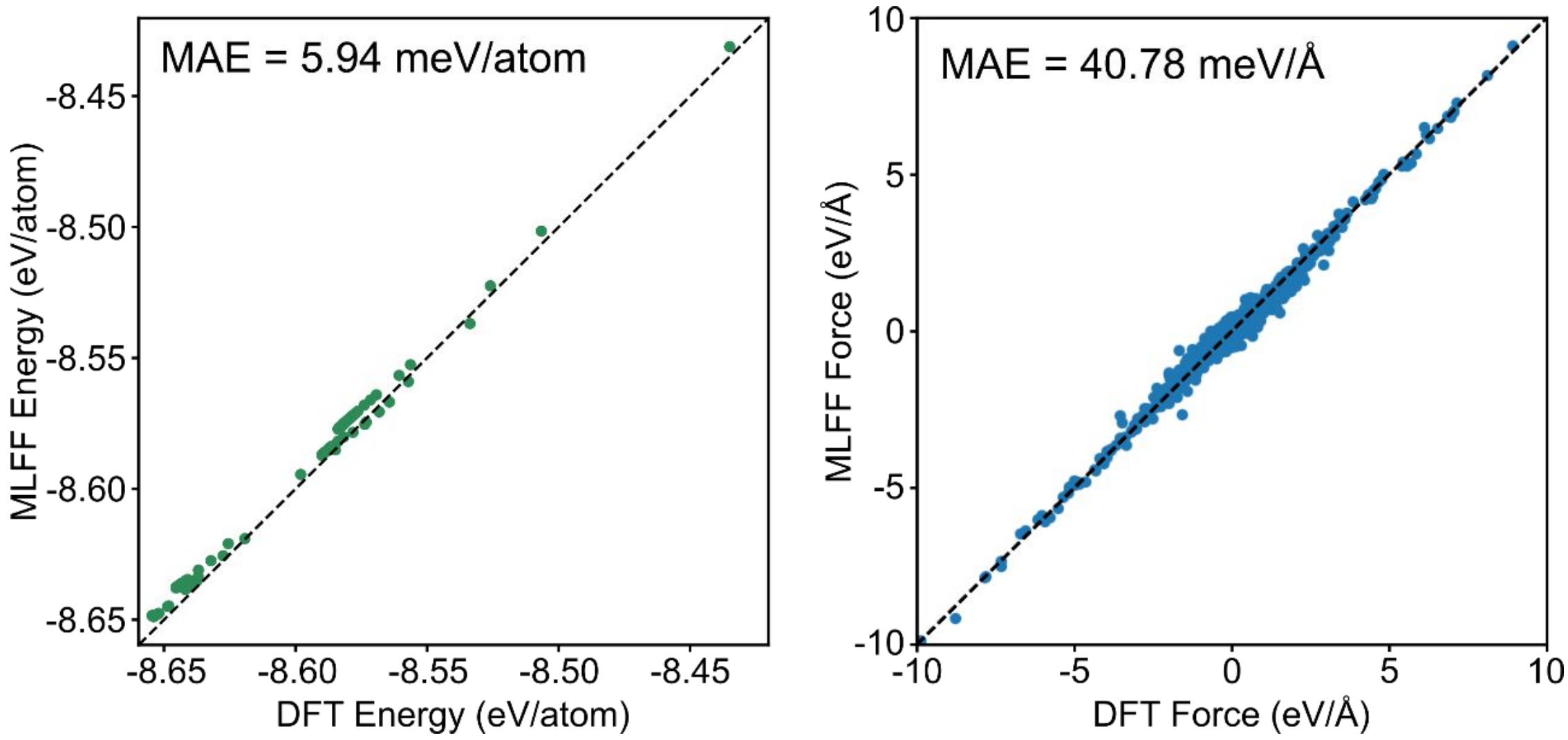

Figure S4. Parity plots between MLFF-predicted and DFT-calculated energies per atom and atomic forces of modified M-N_4 SAC system. In this prediction, UMA with OC20 task is used as MLFF.

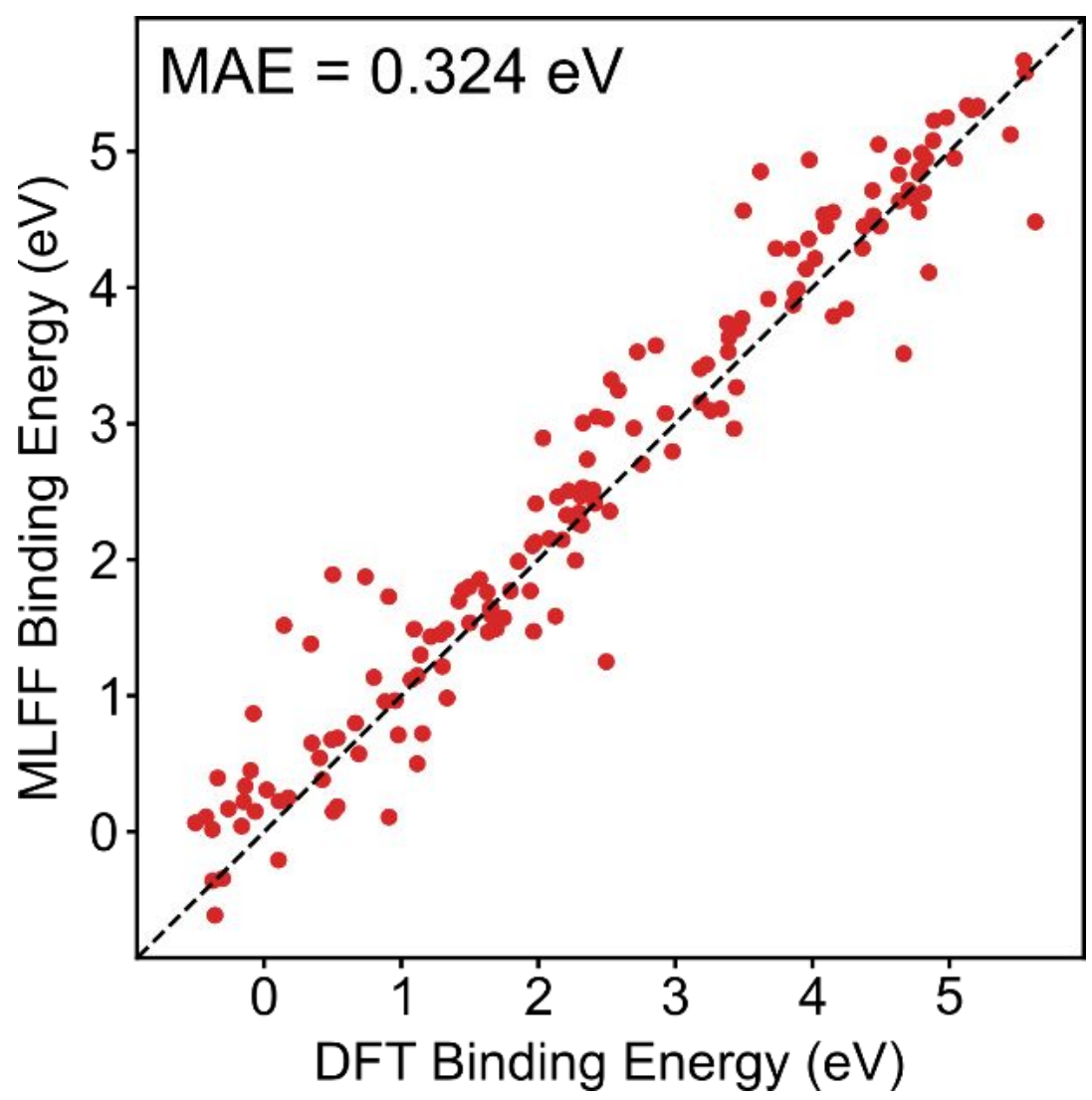

Figure S5. Parity plots between MLFF-predicted and DFT-calculated binding energies of M-N_4 SAC system with various local modifications. Modifications include metal site, 1st shell, 2nd shell substitution, defect and addition of ligand and functional group.

B.3 Pre-validation of LLM

In most cases, the modifications proposed by the LLM induced changes in binding energies consistent with both the intended direction and the underlying reasoning provided by the model. Nevertheless, a limited number of discrepancies were observed. The first type of inconsistency arises from structural distortions driven by local environment effects, as illustrated in **Figure S6a**. In this case, a functional group located in the second shell migrated

toward the metal center during MLFF-optimization. This migration destabilized the bare catalyst structure, increasing its DFT total energy and consequently leading to a reduction in the binding energy. Although the LLM correctly anticipated the change in electron density, it failed to predict binding energy trend due to the unforeseen structural rearrangement.

A second source of inconsistency originates from incorrect analogies to prior cases, as shown in **Figure S6b**. Even when identical modifications are applied, the resulting binding energy trends can differ depending on the current condition of catalyst. In such instances, the LLM neglected this contextual dependence, leading to erroneous predictions for both binding energy and electronic density changes.

To mitigate these limitations within the proposed framework, we introduced additional mechanisms to inform the LLM when substantial structural rearrangements occur after geometry optimization, such as changes in binding configurations. Furthermore, modifications were recorded in the design history together with updated textual descriptions of the catalyst structures. This approach enables the LLM to associate binding energy trends with both the catalyst environment and the applied modification, thereby improving its contextual reasoning in subsequent design iterations.

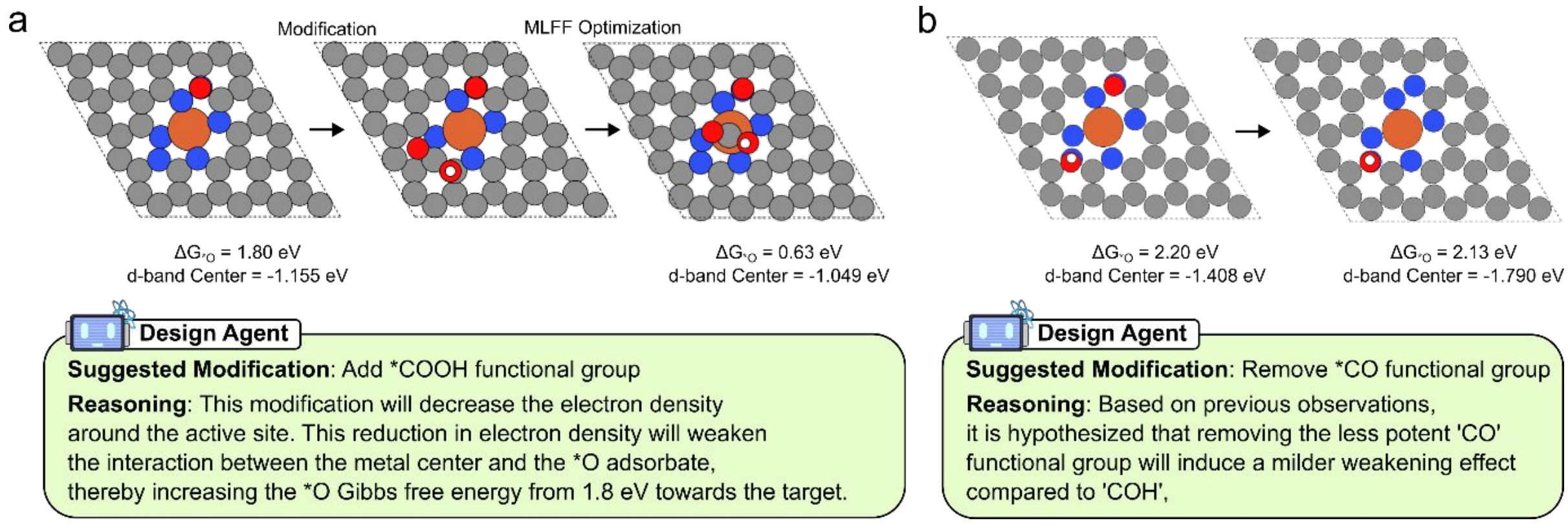

Figure S6. Examples of discrepancies between reasoning of the design agent and calculated results. (a) Case of binding site change due to movement of functional group. (b) Case of incorrect reference to previous observation.

Exploration Summary Report

1) Binding Rule
- Binding_Strength = – ΔG, meaning more positive ΔG values correspond to weaker binding
and more negative ΔG values correspond to stronger binding.

2) Executive Findings
- First-shell coordination substitutions (especially N→O, N→S, O→C/S) effectively tune adsorption Gibbs energies,
but often cause overcorrections, especially for *OOH.
- Second-shell substitutions (notably C↔N, C↔B, B↔P, S↔P) modulate *O and *OH binding more selectively
with smaller effects on *OOH but tend to shift binding strengths insufficiently or excessively depending on direction.
- Metal center substitution significantly affects adsorption energies: Ni and Co yield moderate tuning;
Fe, Mn, Mo, Ti, Ru often cause strong deviations from target ΔG windows and degrade performance.
- Ligand addition (axial O) strongly disrupts binding energies, generally reducing catalyst performance
due to excessive electronic effects.
- Complexity grows moderately with multiple second-shell substitutions;
no major structural distortions were observed across modifications, supporting chemical plausibility.
- Overpotential and dissolution potential trends confirm that balanced tuning of all three adsorbates
remains challenging; many modifications improve one or two ΔGs but leave the others off-target.

3) Modification → Outcome Map
- **First-shell Coordination Substitution:**
  - Pattern: Substitution of first-shell N or O ligands by O or S generally weakens binding (increases ΔG),
improving overpotential but occasionally over-weakening *OOH or strengthening *O/*OH unexpectedly.
  - Examples: Iteration 1 (N→O on Cu; ΔG increases, overpotential ↓), iteration 3 (N→S on Cu; similar weakening),
iteration 20 (O→C on Co; ΔG increase but overshoot), iteration 49 (N→S on Co; ΔG decrease for *OOH, beneficial).

- **Second-shell Substitution:**
  - Pattern: Substitutions of second-shell C with N, B, or P cause moderate shifts in *O and *OH binding,
sometimes selective but often insufficient or overshoot target ranges;
*OOH ΔG less affected but can increase undesirably.
  - Examples: Iteration 2 (C→N; lowers ΔG), iteration 7 (N→P; large overbinding),
iteration 33 (P→B; improves *O and *OH), iteration 39 (C→P; modest weakening but worsened *OOH).

- **Metal Substitution:**
  - Pattern: Ni and Co exhibit moderate, somewhat tunable adsorption energies;
Fe, Mn, Mo, Ti, Ru, Pd and Sn tend to cause large deviations (over-/under-binding) and degrade catalytic metrics.
  - Examples: Iteration 4 (Cu→Ni; moderate tuning), iteration 9 (Ni→Co; stronger binding),
iteration 19 (Co→Mn; too strong binding), iteration 28 (Co→Ti; overbinding),
iteration 35 (Co→Ir; excessive weakening).

- **Ligand Addition:**
  - Pattern: Addition of axial O ligand strongly perturbs binding energies,
typically over-weakening *OOH and excessively strengthening *OH, degrading performance.
  - Example: Iteration 45 (ligand O addition on Co; large ΔG shifts, high overpotential).

4) Selective Adsorbate-Tuning Patterns
- **Substitutions that primarily weaken *OOH binding while strengthening *O and *OH:**
  - Iteration 36 (S→O coordination on Co): *OOH ΔG ↑ (weakened), *O and *OH ΔG ↓ (strengthened).

- **Substitutions that increase *O and *OH binding with minimal *OOH change:**
  - Iteration 33 (P→B in second shell): *O and *OH ΔG increase moderately;
*OOH ΔG increase above target slightly; improves overpotential.

- **Substitutions that weaken *O and *OH binding noticeably with minor effect on *OOH:**
  - Iteration 21 (Co→Ni): *O ΔG closer to target; *OH still low; *OOH nearly unchanged.

- **Substitutions causing simultaneous over-weakening or over-strengthening of all adsorbates:**
  - Iteration 7 (N→P second shell): strong over-binding lowering all ΔG values well below target.
  - Iteration 24 (Co→V metal substitution): large over-binding with strongly negative *O and *OH ΔG.

5) Exploitation Playbook
- Safe moves:
  - First-shell substitutions of N with O or S on Co and Cu centers to moderately weaken *OOH
and reduce overpotential (e.g., Iterations 1, 3, 49).
  - Second-shell substitution of C with B or P on Co to fine-tune *O and *OH binding (Iterations 33, 39).
  - Metal substitution between Co and Ni for moderate tuning without destabilizing structures (Iter.4, 9, 15).
  - Avoid ligand addition unless further refined due to high risk of destabilizing adsorption energetics.
- Conditional moves:
  - Defect or ligand introduction only if complexity margin allows (>4)
and previous binding energies are within acceptable windows, due to risks seen (Ligand O, Iter.45).
  - Explore second-shell heteroatom substitutions (N, P, S) in a controlled sequence to target incremental tuning,
avoiding large jumps (e.g., iterations 7, 18, 39).
  - Test metal substitutions to Ru or Ir only if aim is to explore weaker binding extremes
and stability margins (iterations 35, 42).
- Avoid/guardrails:
  - Excessive metal substitutions to Mo, Ti, V, Mn lead to major overbinding or underbinding
and poor overpotential (iterations 24, 28, 50, 31). Avoid unless different coordination is also explored.
  - First-shell O→C substitutions can cause strong over-weakening
and binding site changes leading to unbalanced energies (Iter.6, 20).
  - Second-shell substitution leading to overshooting *OOH binding outside target range
should be avoided or compensated.
  - Complexities beyond 6 showed only marginal gains and potential synthesis challenges;
cap complexity growth at 6 for exploitation.
- Recurring failure mode: Significant variations in binding site and adsorption geometry
after first-shell substitutions involving heavier atoms (e.g., C, S) or metal changes lead to unpredicted ΔG shifts.
Guard by monitoring structural binding site integrity visually
and rejecting drastic binding site relocations during exploitation.

Summary: Exploitation should prioritize incremental, charge-consistent substitutions
in first and second coordination shells primarily on Co and Ni metals,
avoid severe changes in adsorption geometry or ligand environment,
and carefully balance binding energies of *OOH, *O, and *OH recognizing their differential sensitivities.
Avoid high-complexity designs and metal substitutions with Mo, Ti, and V
unless accompanied with new coordination motifs.
This strategy maximizes likelihood of balanced ORR catalytic performance within accessible complexity limits.

Figure S7. An example of an exploration summary report from the exploration report agent.

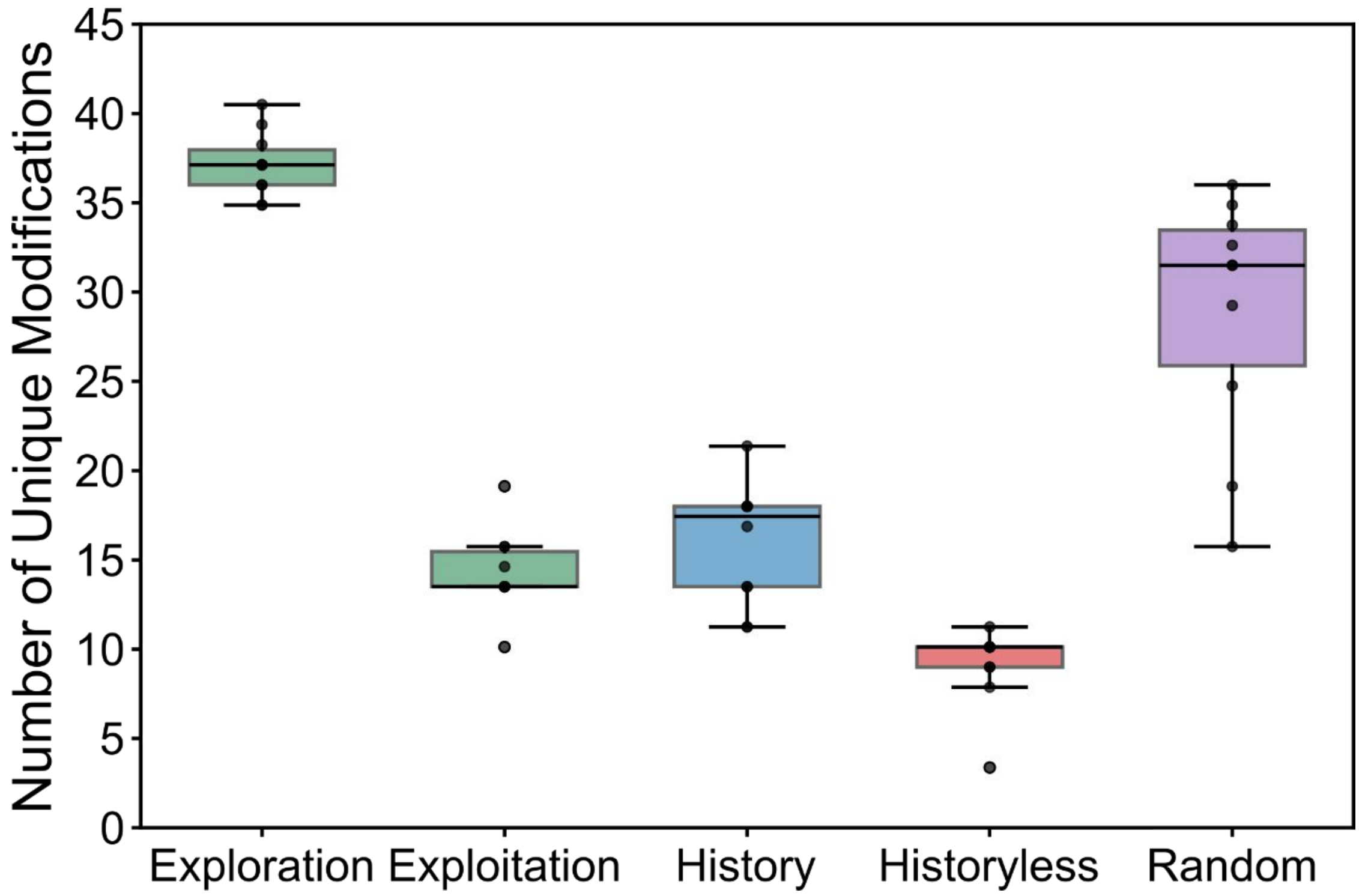

Figure S8. Number of unique modifications proposed during the 50 design steps, averaged over 10 independent design runs. The labels "Exploration" and "Exploitation" correspond to the exploration and exploitation phases of "*history + exploration*" strategy, respectively.

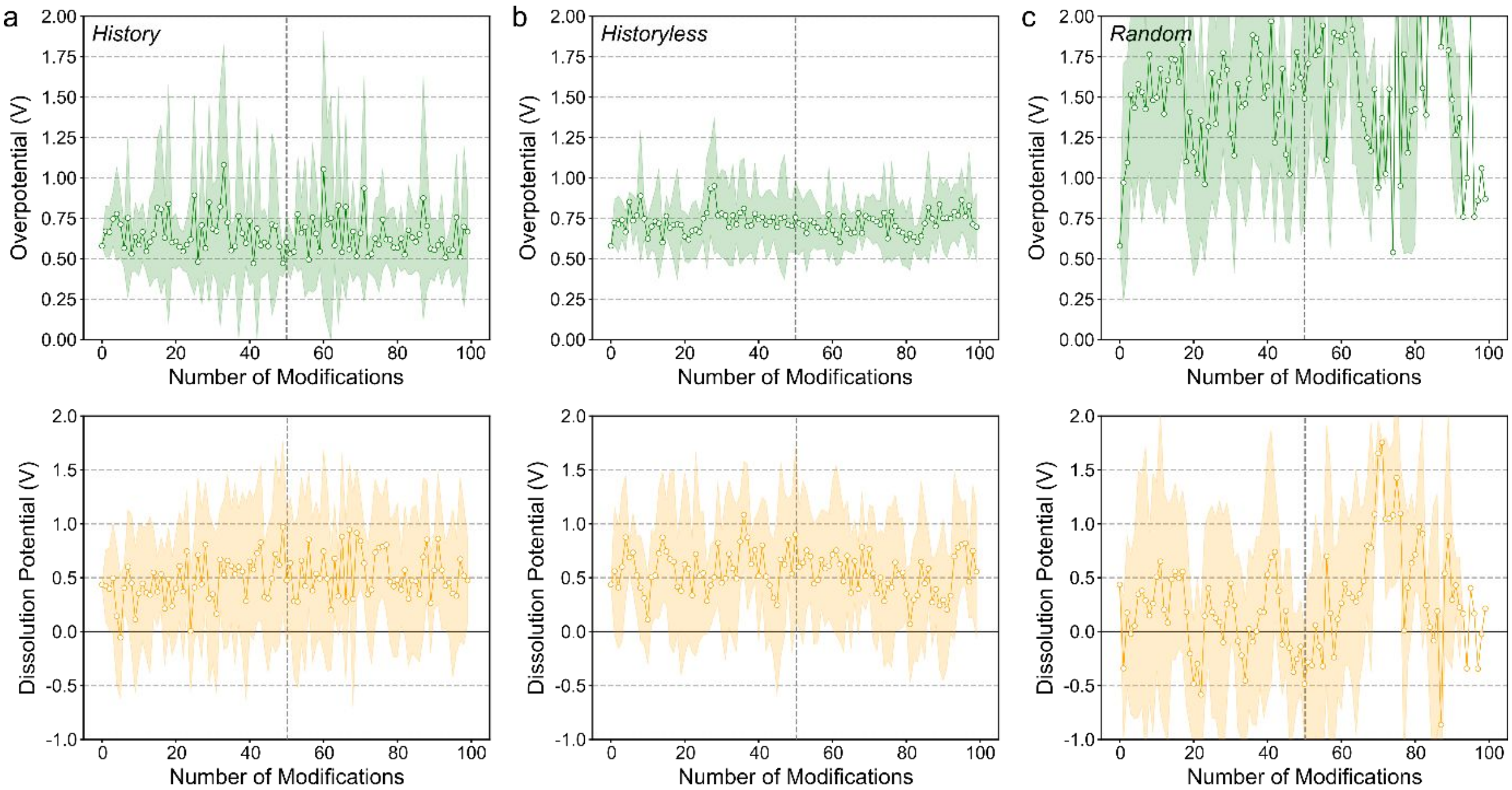

Figure S9. Evolution of overpotential and dissolution potential averaged over 10 independent design runs using (a) "*history*", (b) "*historyless*" and (c) "*random*" strategies.

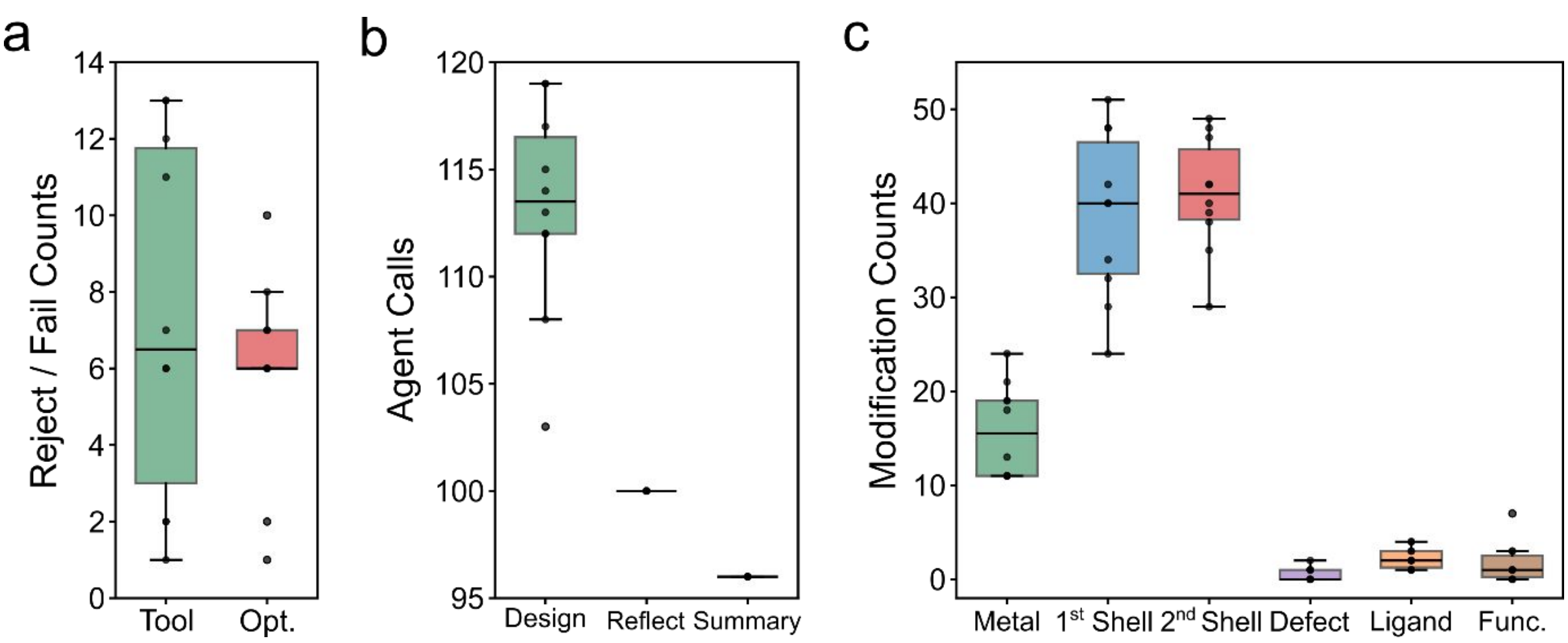

Figure S10. Statistics related to the design loop. (a) Number of rejected modifications caused by invalid response formats or hallucinated outputs (Tool) and the number of failed structure optimizations (Opt.). (b) Number of calls to the design, reflection, and summary agents during the design loop. (c) Number of modifications applied to each geometric site of the SAC structure. All statistics were collected from 10 independent design loops initialized from FeN4 using the "*History + Exploration*" strategy.

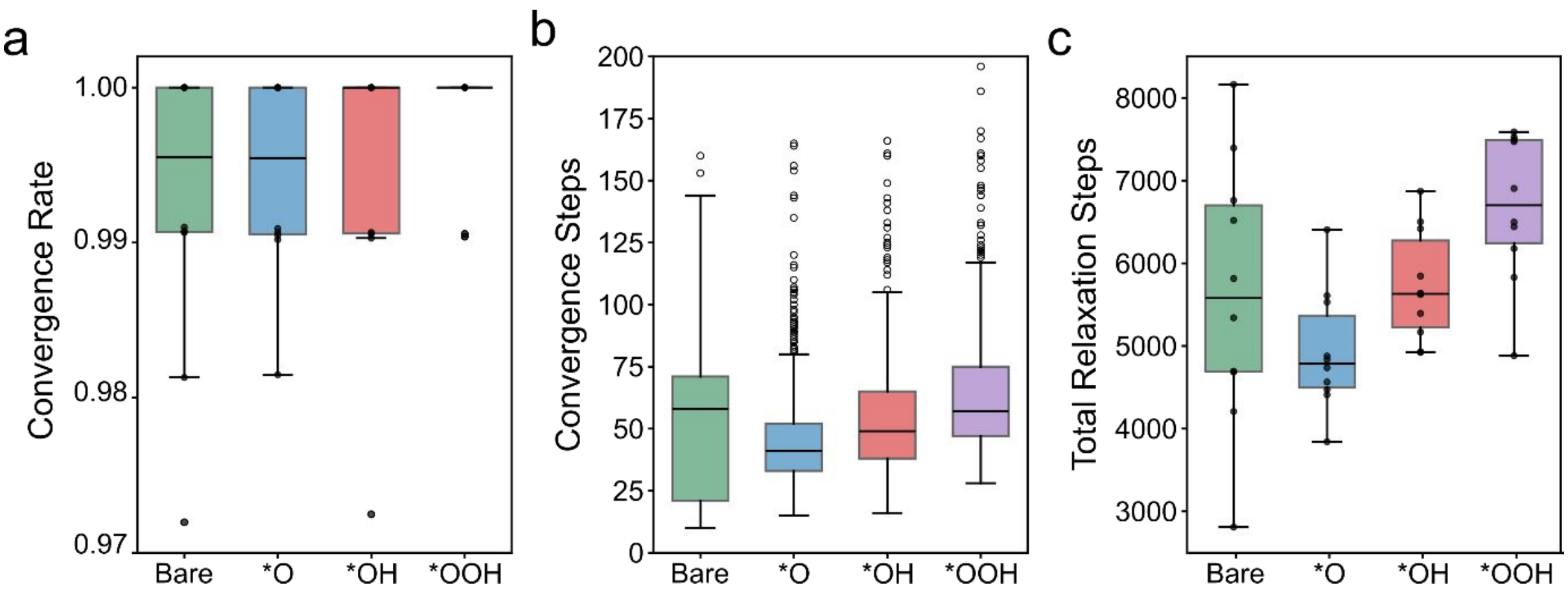

Figure S11. Statistics related to MLFF optimization. (a) Rate of structures that converged within 200 optimization steps using a force threshold of 0.05 eV/Å. (b) Number of optimization steps required for successfully optimized structures to reach the force convergence criterion. (c) Total number of optimization steps consumed during each complete design loop. All optimizations were performed using UMA, and the statistics were collected from 10 independent design loops initialized from FeN4 using the "*History + Exploration*" strategy.

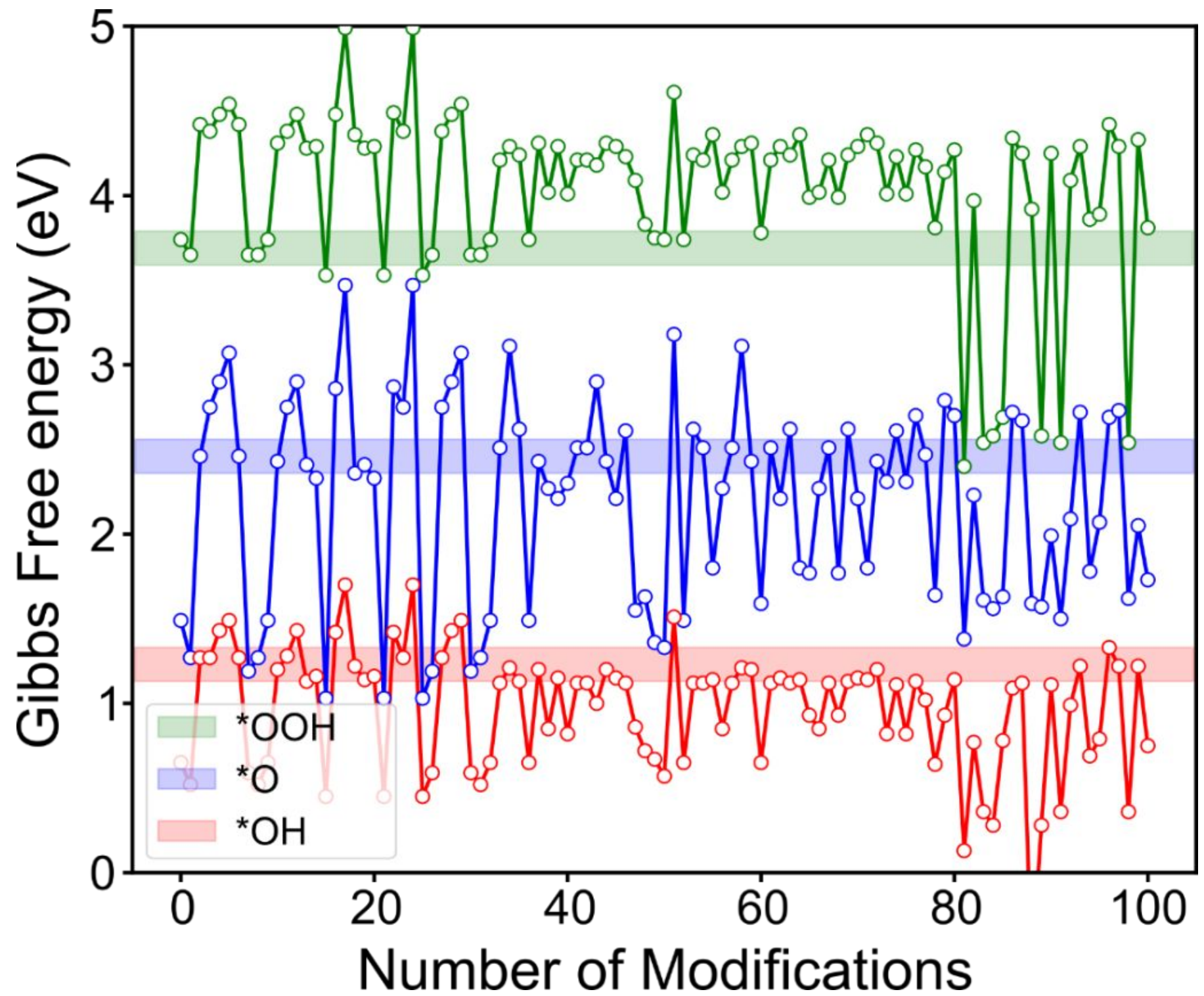

Figure S12. Evolution of the Gibbs free binding energies of *OOH, *O and *OH over 100 design steps from a design run that failed to break the scaling relations. Shaded regions denote the optimal energy windows required to achieve an overpotential below 0.1 V. Because the

binding energies increase and decrease concurrently according to the scaling relations, the three intermediates cannot simultaneously fall within their respective optimal ranges.

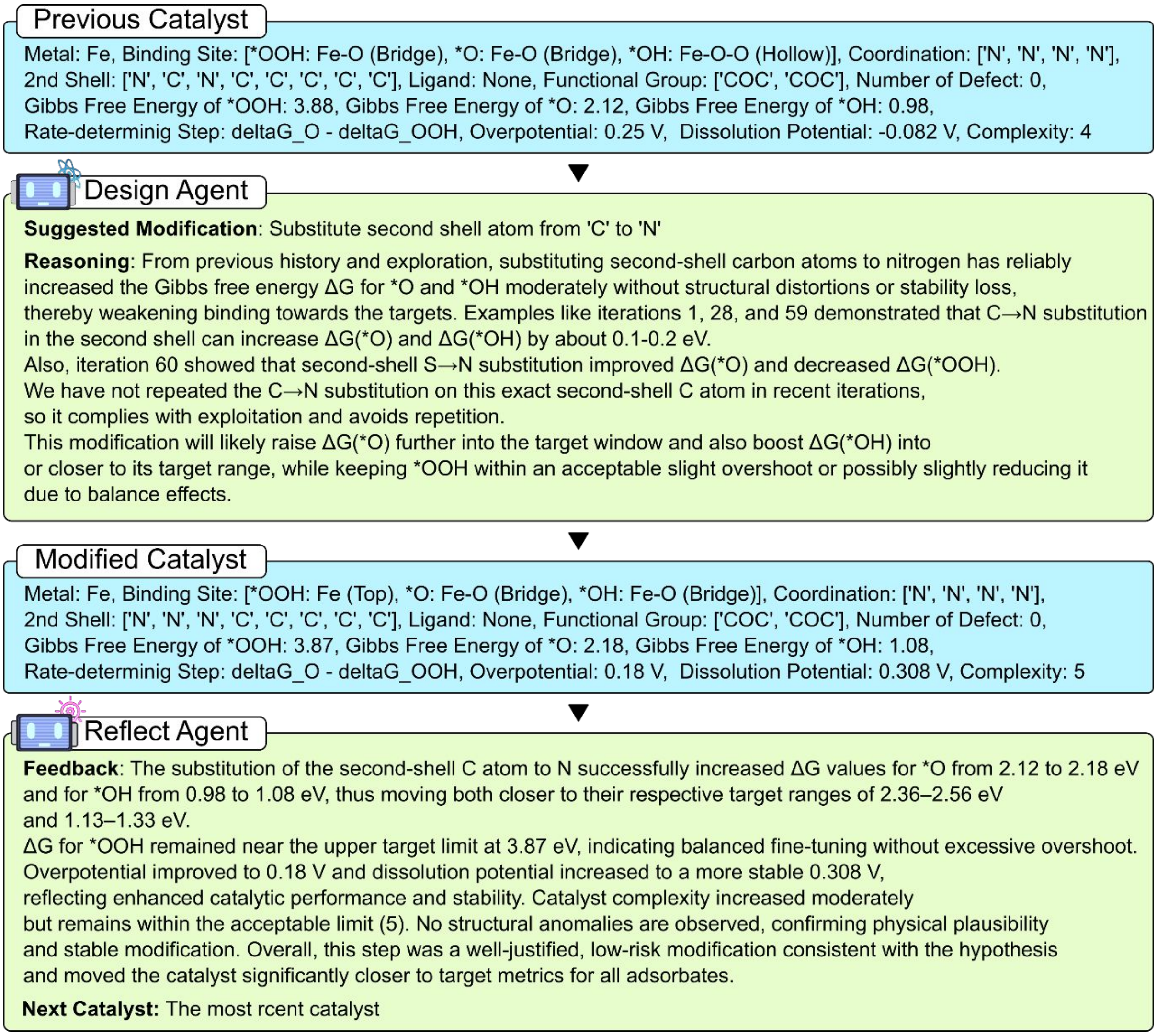

Figure S13. An example of design and self-reflection workflow leading to a minimum overpotential (0.18 V). The design agent suggests a modification by leveraging accumulated design history to selectively tune binding energies. The reflect agent subsequently evaluates modification and calculation results, confirms its effectiveness and provides feedback to guide the next design step.

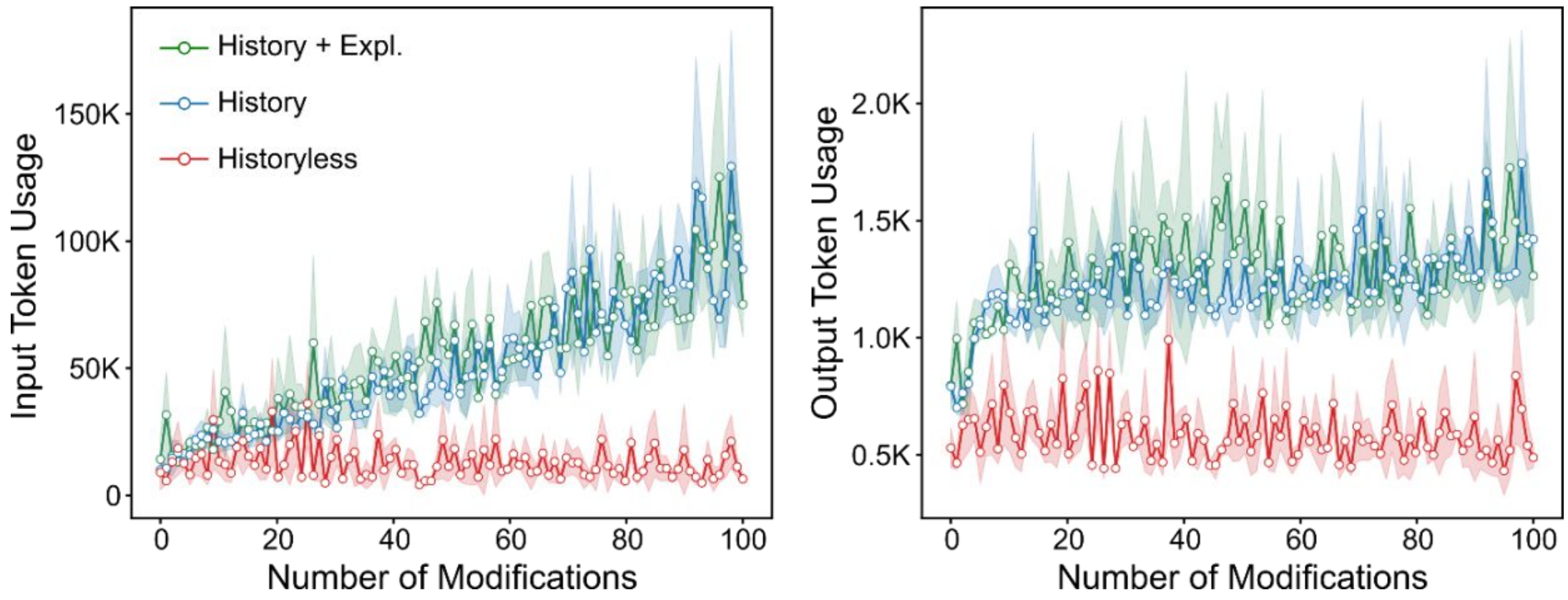

Figure S14. Input and output token usage changes by progress of design run. The *historyless* strategy maintains nearly constant input token usage comparable to the initial value, whereas the *history* and *history + exploration* strategies exhibit a gradual increase in input token usage as design history accumulates.

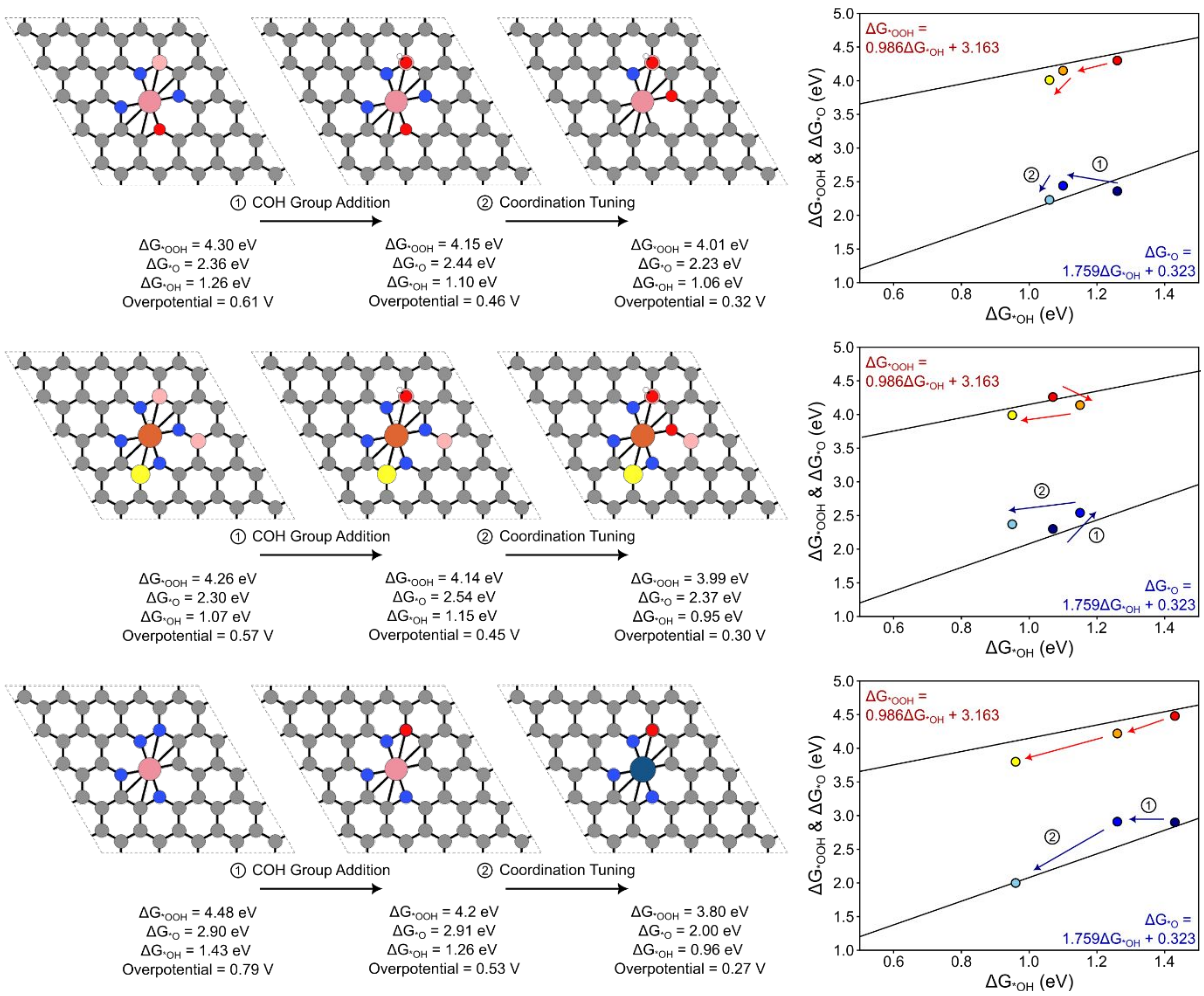

Figure S15. Various representative modification process to design promising catalysts by breaking scaling relations and lower limit of overpotential.

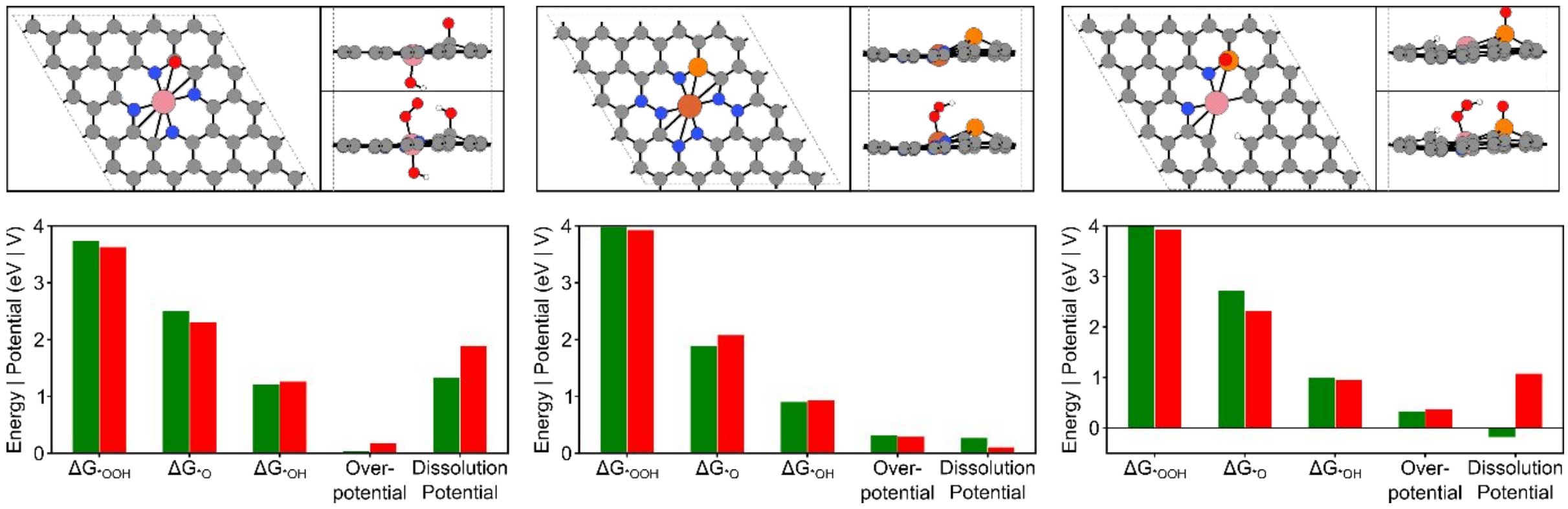

Figure S16. Representation of one of the high-performance catalyst structures discovered during the design runs, together with a comparison of MLFF-predicted and DFT-calculated performance metrics. Data include Gibbs free binding energies, overpotential (η) and dissolution potentials (U_{diss}) for each candidate.

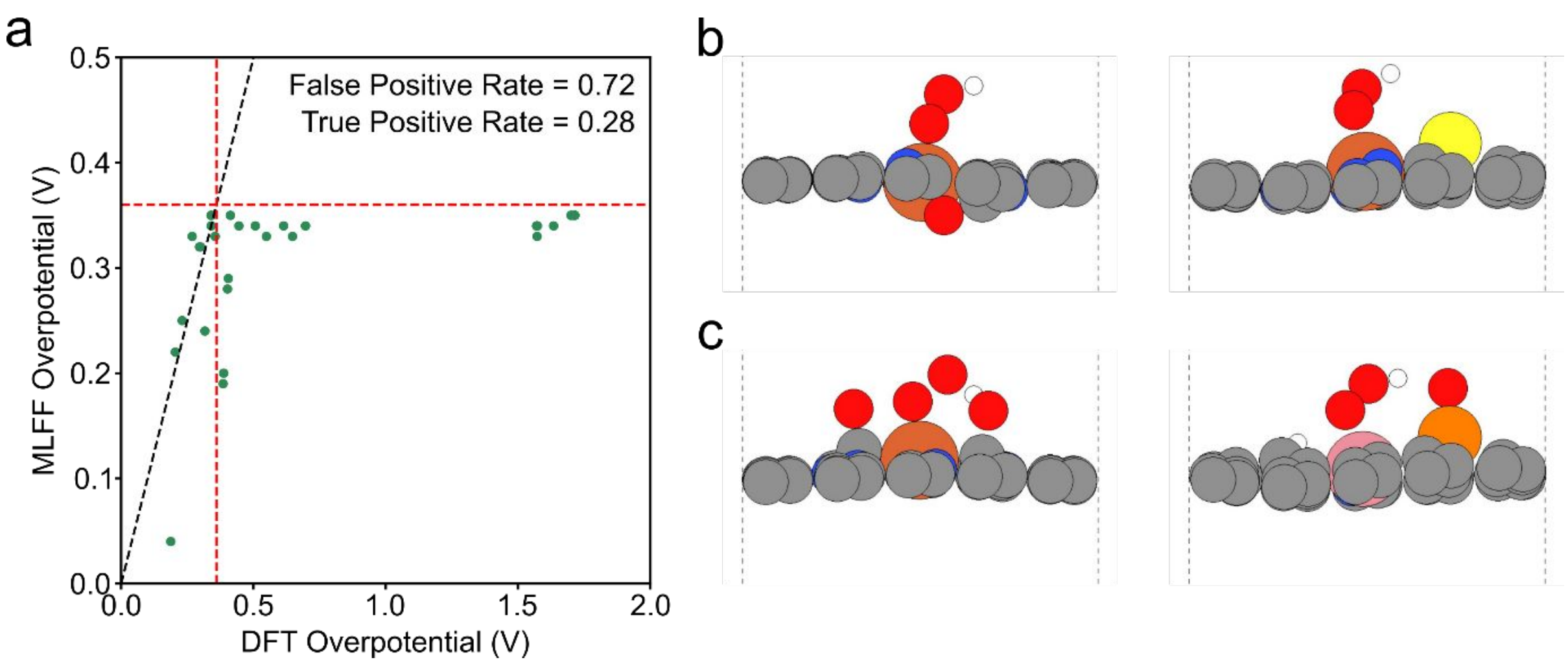

Figure S17. (a) Comparison between MLFF-predicted and DFT-calculated overpotentials of all SAC candidates with MLFF-predicted overpotentials below 0.36 V obtained from 10 independent design runs. The red dashed lines indicate the theoretical overpotential limit of 0.36 V imposed by the scaling relations. (b) Representative false positive candidates. These structures generally lacked H-bonding and exhibited unstable local coordination environments, and (c) Representative true positive candidates. Most of these structures exhibited H-bonding interactions.

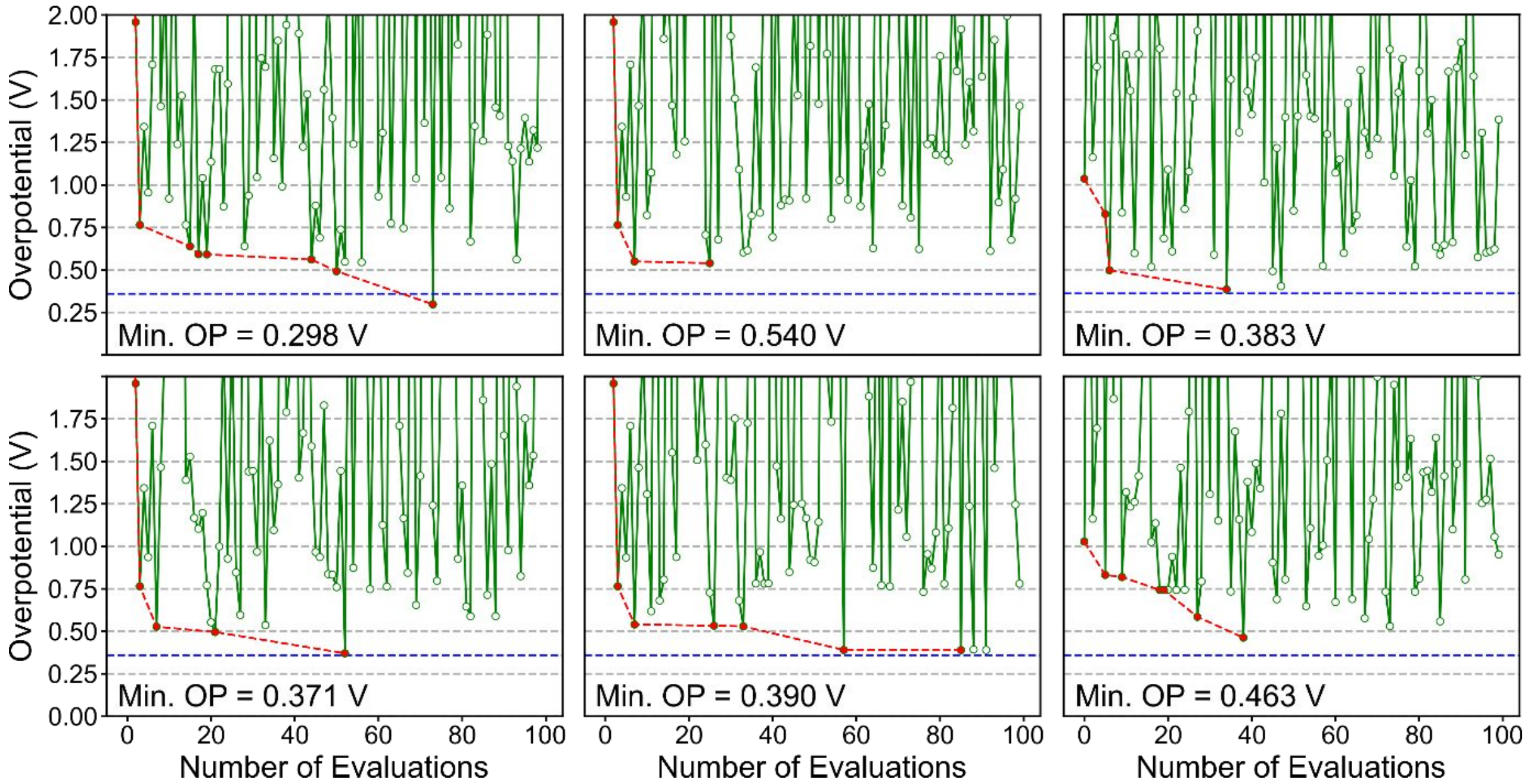

Figure S18. ORR overpotential changes during 6 Bayesian optimization runs. The red and blue dashed lines denote the running minimum overpotential and theoretical minimum overpotential based on scaling relations, respectively.

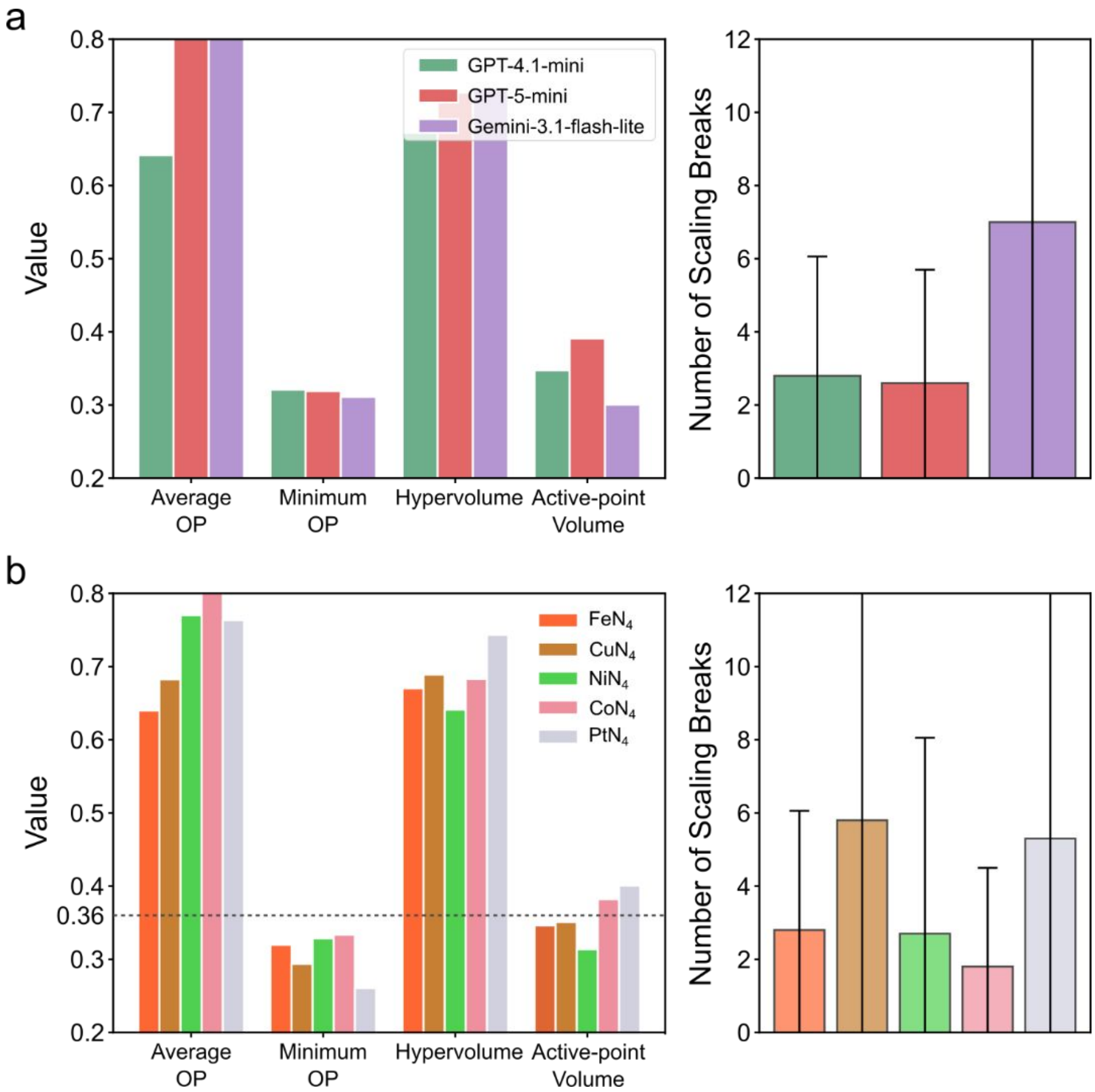

Figure S19. Comparison of overall design performance among (a) different LLMs and (b) different starting SACs. The dashed line indicates the lower limit of overpotential defined by the scaling relations.

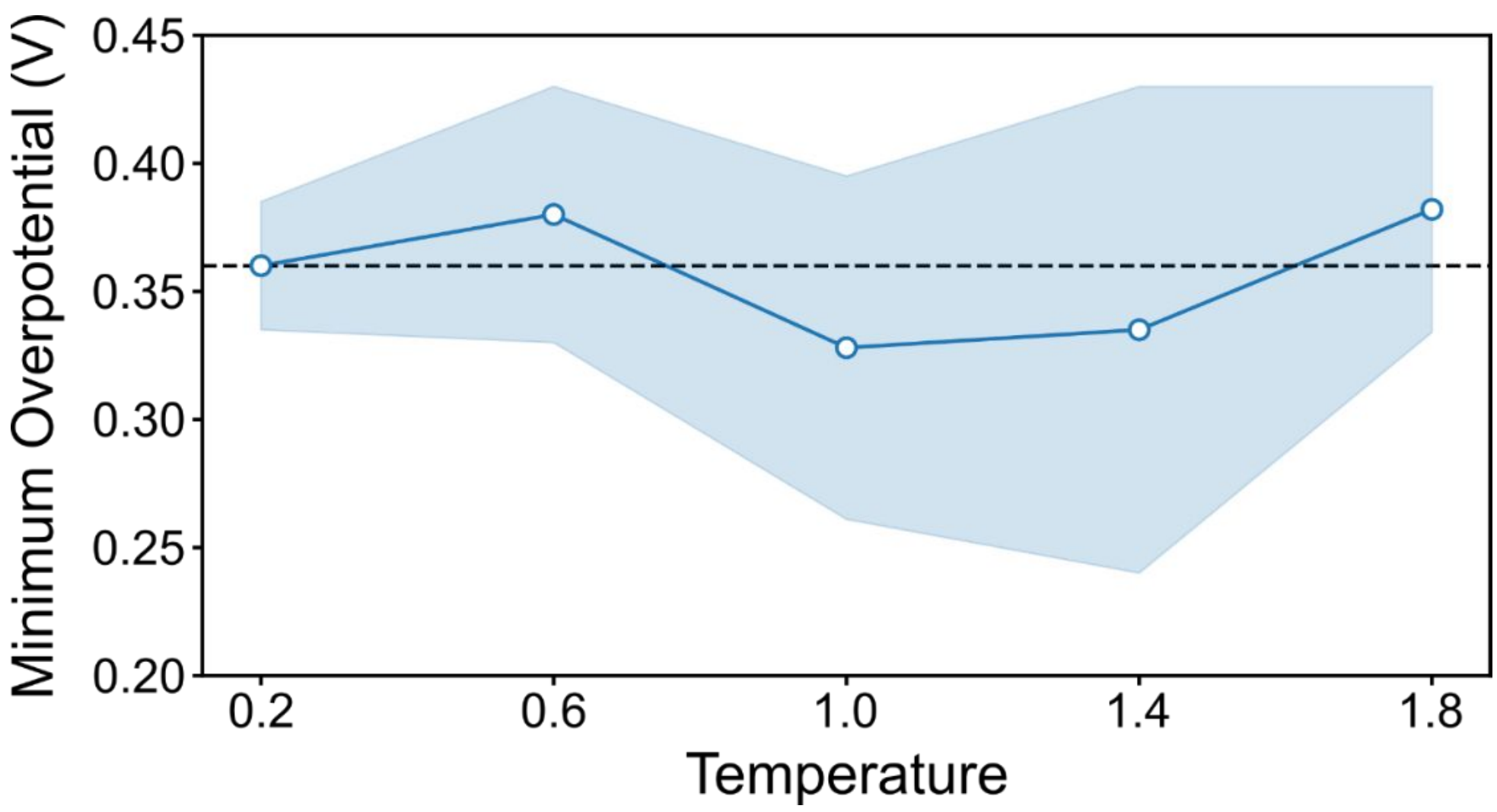

Figure S20. Minimum overpotentials averaged over 10 design runs with each temperature setting. The dashed line indicates lower limit of overpotential defined by the scaling relations.

Table S9. The exploitation-average overpotential (Avg. η), minimum overpotential (Min. η), hypervolume (Hyp. V), Active-point volume (Act. V) and the number of scaling breaks (Break) of 10 independent design runs using "*History + Exploration*" strategy.

Metric	1	2	3	4	5	6	7	8	9	10	Avg.
Avg. η	0.558	0.589	0.689	0.682	0.767	0.803	0.68	0.557	0.558	0.584	0.647
Min. η	0.32	0.28	0.33	0.37	0.43	0.4	0.34	0.33	0.18	0.29	0.327
Hyp.V	0.731	0.618	0.697	0.619	0.696	0.707	0.567	0.627	0.600	0.689	0.655
Act. V	0.360	0.477	0.266	0.202	0.280	0.520	0.385	0.205	0.382	0.424	0.350
Break	7	2	2	0	0	0	5	2	8	4	3.0

Table S10. The metrics of 10 independent design runs using "*History*" strategy.

Metric	1	2	3	4	5	6	7	8	9	10	Avg.
Avg. η	0.583	0.599	0.733	0.641	0.624	0.732	0.691	0.605	0.866	0.87	0.694
Min. η	0.38	0.38	0.38	0.34	0.35	0.33	0.38	0.41	0.33	0.35	0.363
Hyp. V	0.655	0.664	0.656	0.565	0.597	0.601	0.664	0.653	0.695	0.687	0.644
Act. V	0.305	0.306	0.305	0.476	0.284	0.476	0.306	0.366	0.621	0.346	0.379
Break	0	0	0	13	6	7	0	0	5	18	4.9

Table S11. The metrics of 10 independent design runs using "*Historyless*" strategy.

Metric	1	2	3	4	5	6	7	8	9	10	Avg.
Avg. η	0.699	0.736	0.674	0.681	0.716	0.722	0.717	0.767	0.827	0.765	0.730
Min. η	0.38	0.48	0.38	0.38	0.04	0.43	0.51	0.51	0.52	0.5	0.413
Hyp. V	0.659	0.640	0.649	0.649	0.727	0.644	0.637	0.529	0.442	0.532	0.611
Act. V	0.306	0.286	0.305	0.320	0.625	0.333	0.346	0.346	0.345	0.459	0.326
Break	0	0	0	0	1	0	0	0	0	0	0.1

Table S12. The metrics of 10 independent design runs using "*Random*" strategy.

Metric	1	2	3	4	5	6	7	8	9	10	Avg.
Avg. η	1.432	1.255	1.471	1.12	1.529	1.744	1.935	1.522	1.804	0.887	1.470
Min. η	0.24	0.35	0.57	0.44	0.44	0.28	0.58	0.45	0.58	0.56	0.449
Hyp. V	0.804	0.565	0.401	0.546	0.637	0.763	0.693	0.636	0.483	0.537	0.634
Act. V	0.209	0.102	0.416	0.421	0.421	0.594	0.364	0.220	0.364	0.342	0.339
Break	2	1	0	0	0	1	0	0	0	0	0.3

Table S13. Pairwise statistical comparison between the "*History + Exploration*" strategy and baseline strategies over 10 independent design runs. Mean Diff. was calculated as the mean value of the "*History + Exploration*" strategy minus that of the corresponding baseline strategy. Statistical significance was evaluated using two-sided Welch's t-tests. The t statistic represents the standardized difference between two group means, df is the Welch–Satterthwaite effective degrees of freedom, and the p-value indicates the probability of obtaining an equal or more extreme difference under the null hypothesis that the two strategies have the same mean.

Metric	Comparison	Mean Diff.	t	df	p-value
Avg. η	History	−0.048	−1.083	17.60	0.239
	Historyless	−0.084	−2.603	13.32	0.022
	Random	−0.823	−7.836	10.44	0.001
Min. η	History	−0.036	−1.533	11.61	0.152
	Historyless	−0.086	−1.708	12.99	0.111
	Random	−0.122	−2.679	13.97	0.018
# Break	History	−1.900	−0.860	12.61	0.406
	Historyless	2.900	3.137	9.21	0.012
	Random	2.600	2.751	10.04	0.020

Table S14. One-way ANOVA results assessing whether each metric differs significantly among the four design strategies over 10 independent runs. The F statistic is the ratio of between-strategy variance to within-strategy variance and the p-value represents the probability of obtaining an equal or larger F statistic under the null hypothesis that all strategy means are identical.

Metric	One-way ANOVA F	ANOVA p-value
Avg. η	49.52	7.38×10^{-13}
Min. η	2.76	0.0563
# Break	4.20	0.0120

Table S15. DFT-calculated, MLFF-predicted overpotential and *H atom Bader charge of SAC candidates with MLFF-predicted overpotentials below 0.36 V identified across 10 independent design runs for each strategy. "Exp" denotes the "*History + Exploration*" strategy, "His" denotes the "*History*" strategy, and "No His" denotes the "*Historyless*" strategy. Bold values indicate true positive candidates for which both the MLFF-predicted and DFT-calculated overpotentials are below 0.36 V. *H atom Bader charges are evaluated from *OOH adsorbate.

Strategy	Run	Iter.	DFT OP (V)	MLFF OP (V)	Bader (e)	Strategy	Run	Iter.	DFT OP (V)	MLFF OP (V)	Bader (e)
Exp	1	55	1.572	0.34	0.565	Exp	4	51	0.387	0.20	0.608
		57	2.230	0.34	0.599			62	0.385	0.19	0.608
		60	2.620	0.35	0.556	Exp	6	**85**	**0.230**	**0.25**	**0.624**
		87	2.735	0.35	0.580			**86**	**0.316**	**0.24**	**0.639**
		90	1.634	0.34	0.584			**100**	**0.205**	**0.22**	**0.632**
		93	0.549	0.33	0.610	Exp	8	63	0.445	0.34	0.564
		**97**	**0.355**	**0.33**	**0.646**			**67**	**0.296**	**0.32**	**0.581**
Exp	9	82	0.402	0.28	0.604			**68**	**0.341**	**0.35**	**0.569**
		91	0.405	0.29	0.605			73	0.507	0.34	0.570
Exp	10	**31**	**0.268**	**0.33**	**0.617**			**74**	**0.300**	**0.32**	**0.601**
		35	0.614	0.34	0.595	His	4	10	1.571	0.34	0.561
His	5	**10**	**0.340**	**0.35**	**0.574**			11	1.714	0.35	0.580
		41	1.701	0.35	0.563			12	1.174	0.35	0.578
		95	1.701	0.35	0.569			24	1.573	0.34	0.560
His	6	43	1.570	0.34	0.560			**39**	**0.340**	**0.35**	**0.568**
		49	1.572	0.33	0.559			45	1.713	0.35	0.575
		**61**	**0.340**	**0.34**	**0.569**			46	3.690	0.34	0.556
		69	1.714	0.35	0.578	His	9	13	2.621	0.34	0.555
		77	1.714	0.35	0.569			14	2.230	0.35	0.561
		84	1.741	0.35	0.582			19	2.620	0.33	0.555
His	10	49	0.413	0.35	0.571			26	0.647	0.34	0.588
No His	9	**53**	**0.187**	**0.04**	**0.612**			97	0.697	0.35	0.579

Table S16. The Gibbs free energy correction values for adsorbates and gaseous molecules. All units are in eV.

Adsorbates	ZPE	$\int C_p dT$	-TS	Molecules	ZPE	$\int C_p dT$	-TS
O*	0.08	0.03	-0.06	H_2	0.28	0.09	-0.41
OH*	0.37	0.04	-0.07	H_2O	0.57	0.10	-0.67
OOH*	0.44	0.05	-0.09				

Reference

(1) Jia, S.; Varma, A.; Manivannan, P.; Chayapathy, D.; Fung, V. Benchmarking Text Representations for Crystal Structure Generation with Large Language Models. In *AI for Accelerated Materials Design-ICLR 2025*.
